# Endothelial c-REL orchestrates atherosclerosis at regions of disturbed flow through crosstalk with TXNIP-p38 and non-canonical NF-κB pathways

**DOI:** 10.1093/cvr/cvaf024

**Published:** 2025-02-21

**Authors:** Blanca Tardajos Ayllon, Neil Bowden, Celine Souilhol, Hazem Darwish, Siyu Tian, Carrie Duckworth, David Mark Pritchard, Suowen Xu, Jon Sayers, Sheila Francis, Jovana Serbanovic-Canic, Fiona Oakley, Paul Charles Evans

**Affiliations:** William Harvey Research Institute, Barts and The London School of Medicine and Dentistry, Queen Mary University of London, London EC1M 6BQ, UK; School of Medicine and Population Health, INSIGNEO Institute, and the Bateson Centre, University of Sheffield, Sheffield, UK; School of Medicine and Population Health, INSIGNEO Institute, and the Bateson Centre, University of Sheffield, Sheffield, UK; School of Medicine and Population Health, INSIGNEO Institute, and the Bateson Centre, University of Sheffield, Sheffield, UK; School of Medicine and Population Health, INSIGNEO Institute, and the Bateson Centre, University of Sheffield, Sheffield, UK; William Harvey Research Institute, Barts and The London School of Medicine and Dentistry, Queen Mary University of London, London EC1M 6BQ, UK; School of Medicine and Population Health, INSIGNEO Institute, and the Bateson Centre, University of Sheffield, Sheffield, UK; Department of Molecular and Clinical Cancer Medicine, Institute of Systems, Molecular and Integrative Biology, University of Liverpool, Liverpool, UK; Department of Molecular and Clinical Cancer Medicine, Institute of Systems, Molecular and Integrative Biology, University of Liverpool, Liverpool, UK; Department of Endocrinology, Institute of Endocrine and Metabolic Disease, The First Affiliated Hospital of USTC, Division of Life Sciences and Medicine, Clinical Research Hospital of Chinese Academy of Sciences (Hefei), University of Science and Technology of China, Hefei, China; School of Medicine and Population Health, INSIGNEO Institute, and the Bateson Centre, University of Sheffield, Sheffield, UK; School of Medicine and Population Health, INSIGNEO Institute, and the Bateson Centre, University of Sheffield, Sheffield, UK; School of Medicine and Population Health, INSIGNEO Institute, and the Bateson Centre, University of Sheffield, Sheffield, UK; Bioscience Institute, Faculty of Medical Science, Newcastle University, Newcastle Upon Tyne, UK; William Harvey Research Institute, Barts and The London School of Medicine and Dentistry, Queen Mary University of London, London EC1M 6BQ, UK

**Keywords:** c-REL, Shear stress, Endothelial, Atherosclerosis

## Abstract

**Aims:**

Atherosclerosis initiation at sites of disturbed blood flow involves heightened inflammation coupled to excessive endothelial cell (EC) proliferation. Here, we unveil the pivotal role of c-REL, a member of the NF-κB transcription factor family, in orchestrating these processes by driving dual pathological inflammatory and cell cycle pathways.

**Methods and results:**

Analysis of cultured EC and murine models revealed enrichment and activation of c-REL at atherosusceptible sites experiencing disturbed flow. Transcriptome analysis, extensively validated *in vitro* and *in vivo*, demonstrates that endothelial c-REL drives inflammation via a TXNIP-p38 MAP kinase signalling pathway and enhances proliferation through a non-canonical *NFKB2*-p21 pathway. Consistent with its pivotal role in EC pathology, genetic deletion of *c-Rel* in EC significantly reduces plaque burden in hypercholesterolaemic mice.

**Conclusion:**

These findings underscore the fundamental role of c-REL in endothelial responses to disturbed flow and highlight therapeutic targeting of endothelial c-REL as a potential strategy for atherosclerosis treatment.

## Graphical Abstract

**Graphical Abstract:**
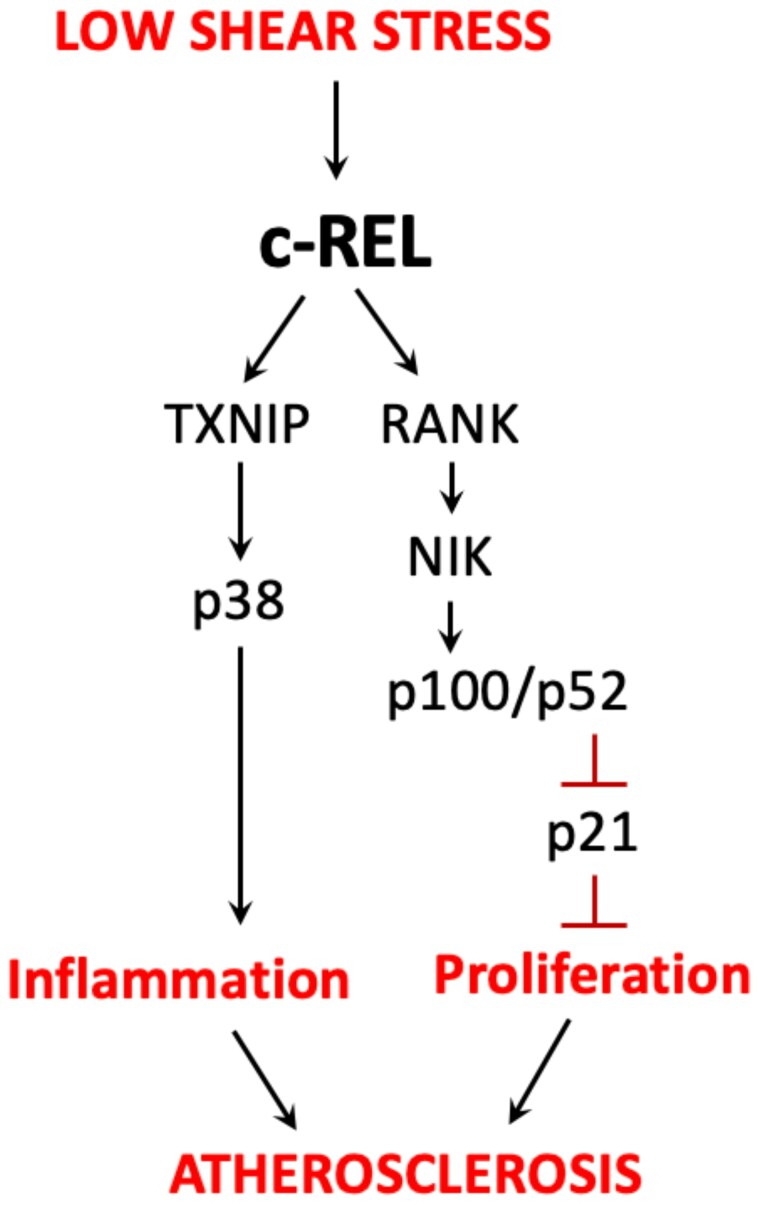

**This manuscript was handled by Guest Editor Thomas F. Lüscher. Time of primary review: 61 daysSee the editorial comment for this article ‘It is all c-RELative: a new perspective for a member of the nuclear factor kappa B family', by J. O’Connor Miranda *et al*., https://doi.org/10.1093/cvr/cvaf053.**

## 1. Introduction

Atherosclerosis, a major cause of mortality, manifests as a focal disease characterized by the accumulation of lipids and inflammatory cells within arterial walls. While systemic risk factors such as hypercholesterolaemia contribute to its onset, atherosclerosis primarily develops near arterial branches and bends exposed to disturbed blood flow.^1–3^ These haemodynamic conditions result in low wall shear stress (WSS), leading to vascular inflammation and heightened proliferation of endothelial cells (ECs).^4–6^ While basal EC proliferation is crucial for vascular repair, excessive proliferation at sites of low shear stress promotes atherosclerosis initiation by increasing arterial permeability to cholesterol-rich lipoproteins.^7^

The NF-κB family of transcription factors comprises five subunits: RELA (*RELA*), RELB (*RELB*), c-REL (*c-REL/REL*), p105/p50 (*NFKB1*), and p100/p52 (*NFKB2*), which form active transcription factors through homo- or heterodimerization.^8^ Extensive research has concentrated on the canonical NF-κB family member, RELA, in the context of atherosclerosis, revealing its regulatory influence on vascular cell function, inflammation, and immunity.^9,10^ Mice with EC-specific deletion of *RelA* exhibit diminished atherosclerosis, underscoring its pivotal role in this disease. Our studies, along with others’, show that ECs at atheroprone sites are predisposed to heightened activation of RELA,^11–14^ offering a mechanistic explanation for increased inflammation in these regions. Despite its significance, RELA functions as a ‘double-edged sword’ in ECs, concurrently activating genes that drive inflammation and an anti-apoptotic transcriptional programme crucial for cell survival.^15–17^ Consequently, targeting RELA for anti-inflammatory therapy encounters cytotoxicity which limits its potential therapeutic use.

Despite the extensive research on RELA in EC activation, the NF-κB family member c-REL possesses distinct biology and is still predominantly unexplored in this context. Here, we identify a role of endothelial c-REL in promoting inflammation and EC proliferation at sites of low shear stress and delineate the involvement of this pathway in atherosclerosis. The mechanism involves thioredoxin-interacting protein (TXNIP)-p38 (*MAPK14*) signalling driving inflammation and a *NFKB2*-p21 pathway driving EC proliferation. Our findings identify endothelial c-REL as a promising therapeutic target, offering a novel strategy to enhance EC function and alleviate atherosclerosis.

## 2. Methods

### 2.1 Experimental animals

All animal care and experimental procedures conformed to the institution’s ethical requirements, UK Home Office regulations, and guidelines from Directive 2010/63/EU of the European Parliament on the protection of animals used for scientific purposes. Animal care and experimental procedures were carried out under Project Licence P5395C858 issued by the UK Home Office. Male mice aged between 2 and 4 months were utilized for experiments. Animals were euthanized through anaesthetic overdose via intraperitoneal (IP) injection of sodium pentobarbital.

#### 2.1.1 Transgenic mice

Mice with conditional deletion of *c-Rel* (*Rel*) in ECs (*c-Rel^ECKO^*) were generated through the crossbreeding of *c-Rel^fl/fl^* mice^18^ kindly provided by Professor Ulf Klein (University of Leeds, UK) with *Cdh5^Cre−ERT2^* mice.^19^ CRE activation was induced by IP administration of tamoxifen (Sigma) in corn oil for five consecutive days (2 mg/mouse/day). Mice with constitutive deletion of *c-Rel* (*c-Rel^KO^*)^20^ or *Nfkb2* (*Nfkb2^KO^*)^21^ were analysed. All transgenic mice were maintained on a C57BL/6J background. Genotyping was performed using PCR primers listed in Supplementary material online, *Table S1*. Two weeks after the first injection of tamoxifen, hypercholesterolaemia was induced by IP injection of adeno-associated virus carrying a gain-of-function mutated version of proprotein convertase subtilisin/kexin type 9 (rAAV8-D377Y-mPCSK9) gene (Vector Core, North Carolina), followed by a western diet (SDS UK, 829100) for 6 weeks, as previously detailed.^22^

#### 2.1.2 Pharmacological inhibition of c-REL in hypercholesterolaemic mice

Hypercholesterolaemia was induced by IP injection of rAAV8-D377Y-mPCSK9 followed by a western diet for 6 weeks. From Weeks 3–6, c-REL was inhibited pharmacologically in wild-type C57BL/6J mice by IP injections of IT603 (24 mg/kg dissolved in 75% DMSO in 0.9% sodium chloride; three injections per week). Control mice received IP injections of 75% DMSO in 0.9% sodium chloride.

### 2.2 Analysis of atherosclerosis plaque

Following euthanasia and perfusion fixation with PBS and 4% paraformaldehyde (PFA), aortas were dissected, cleaned, and stained with Oil Red O (Sigma). Aortas were then imaged through a Stemi 2000 C microscope (Zeiss) using a Canon PowerShot A650 IS digital camera, and lesion areas were quantified using NIS elements analysis software (Nikon, NY).

### 2.3 Plasma lipid measurements

Blood samples collected by terminal cardiac puncture were subjected to centrifugation to obtain plasma. Plasma cholesterol and triglyceride levels were measured using a COBAS analyser.

### 2.4 *En face* staining of murine endothelium

Expression levels of specific proteins in ECs at distinct locations of the murine aortic arch, inner curvature (low shear stress site) and outer curvature (high shear stress site), were assessed by *en face* staining.^23^ Aortas of animals humanely killed by IP injection of pentobarbital were perfusion-fixed with 4% PFA and immunostained using specific antibodies (see Supplementary material online, *Table S2*). Confocal microscopy (Olympus SZ1000 confocal inverted microscope) was utilized to visualize endothelial surfaces *en face*, and mean fluorescence intensities were quantified to determine protein expression levels.

### 2.5 EC culture and exposure to WSS

Human umbilical vein ECs (HUVECs) were isolated from umbilical cords (Royal Hallamshire Hospital, Sheffield, UK; ethical approval REC 10/H1308/25) or purchased from PromoCell and were maintained in M199 growth medium supplemented with 20% (v/v) foetal bovine serum, 100 μg/mL streptomycin, 100 U/mL penicillin, 2.5 μg/mL amphotericin B, 4 mmol/L L-glutamine, 10 U/mL heparin, and 30 μg/mL EC growth supplement. Human coronary artery ECs (HCAECs) were obtained from PromoCell and cultured following the manufacturer’s instructions in supplemented Endothelial Cell Growth Medium MV2. Cells were analysed from individual donors so that variation between individuals could be captured. Cells at passage 3–6 were seeded onto gelatin-coated 6-well plates. When they reached confluency, they were exposed to flow using an orbital shaking platform (PSU-10i; Grant Bio) set at 210 rpm in a 37°C cell culture incubator, which generates low tangential shear stress (4.8 dyn/cm^2^) at the centre of the well and high uniform shear stress (11.1 dyn/cm^2^) at the periphery. For RNA and protein expression analysis, cells were isolated from the centre (circular region, 5 mm radius) or periphery (ring-shaped region, 8 mm wide) of wells by scraping using a standardized template, which is based on the computational fluid dynamics model.^24^

### 2.6 Gene silencing

EC cultures were transfected with siRNA sequences targeting *c-REL* (StealthRNAi siRNA RELHSS109157, Thermo Fisher Scientific) or *NFKB2* (L-003918-00-0005, ON-TARGETplus siRNA, Dharmacon) using the Neon transfection system (Thermo Fisher Scientific; HUVEC) or Lipofectamine RNAiMAX transfection reagent (Thermo Fisher Scientific; HCAEC), following the manufacturer’s instructions. Non-targeting scrambled sequences served as a control (D-001810-01-50 ON-TARGETplus Non-targeting siRNA, Dharmacon).

### 2.7 Lentiviral-mediated gene expression

Plasmids expressing FLAG-tagged versions of c-REL, p38, and TXNIP were synthesized and sub-cloned into the pGenLenti vector (GenScript Biotech). To produce lentivirus, pGenLenti vectors containing *c-REL*, *p38*, or *TXNIP* cDNA were transfected into HEK/293T cells together with the second-generation lentiviral packaging plasmid (psPAX2; Addgene) and the envelope plasmid (pMD2.G; Addgene) and cultured in Opti-MEM medium using polyethyleneimine (PEI), at a PEI/DNA ratio of 3:1. Virus was collected from medium 48–72 h after transfection.

### 2.8 Bulk RNA analysis and qRT-PCR

ECs were isolated from the centre of 4 wells of a 6-well plate and pooled prior to bulk RNA analysis. RNA was extracted using the RNeasy Mini Kit (74104, Qiagen) and reverse transcribed into cDNA using the iScript cDNA synthesis kit (1708891, Bio-Rad). Bulk RNA analysis was carried out using the Human Clariom™ S Assay (Affymetrix), and data were analysed using Transcriptome Analysis Console Software (Affymetrix). Functional enrichments for protein-coding genes with *P* < 0.05 and log2 fold change > 1.2 were calculated using DAVID Functional Annotation Bioinformatics Microarray Analysis Software^25^ and Metascape Software.^26^ ECs were isolated from the centre of 2 wells of a 6-well plate prior to qRT-PCR analysis with gene-specific primers (see Supplementary material online, *Table S3*). Reactions were prepared using SsoAdvanced universal SYBR®Green supermix (172-5271, Bio-Rad) and following the manufacturer’s instructions and were performed in triplicate. Expression values were normalized against the house-keeping gene *HPRT*. Data were pooled from at least three independent donors, and mean values were calculated with SEM.

### 2.9 Immunofluorescent staining of cultured EC

ECs were fixed, permeabilized, and blocked with goat serum as described previously. They were incubated overnight with primary antibodies against PCNA or Ki67 (proliferation markers); Caspase3 (apoptosis marker); p21, p53, or H2AX (senescence markers); and NF-κB subunits (see Supplementary material online, *Table S2*) and Alexa Fluor 488 or 568-conjugated secondary antibodies. Nuclei were identified using DAPI (Sigma). Images were taken with a widefield fluorescence microscope (LeicaDMI4000B) and analysed using ImageJ software (1.49p) to calculate the frequency of positive cells. Isotype controls or omission of the primary antibody was used to control for non-specific staining.

### 2.10 Immunoblotting

Total cell lysates were prepared using lysis buffer, and immunoblotting was conducted using specified primary antibodies (see Supplementary material online, *Table S2*) and horseradish peroxidase–conjugated secondary antibodies. Chemiluminescent detection was performed using ECL Prime® (GE Healthcare), and membranes were imaged with the Gel Doc XR + system (Bio-Rad). Alternatively, membranes were exposed to film (GE Healthcare) in a dark room, and the film was developed using developing solution (Ilford PQ Universal) and fixed with Ilford Hypam fixer.

### 2.11 Generation of purified c-REL

Human *c-REL* cDNA (NP_002899.1) was cloned into the pET-21a(+) expression vector using standard restriction enzyme cloning techniques. *Escherichia coli* [BL21(DE3)] containing pET-21a(+)-*c-REL* were cultured using autoinduction LB media at 20°C with shaking for 36 h. Bacterial pellets were collected and lysed in Tris-HCl buffer (pH 7) containing lysozyme followed by sonication. The recombinant c-REL protein was purified from the bacterial lysate through ion exchange followed by gel filtration chromatography.

### 2.12 Electrophoretic mobility shift assay

A fluorescent (3′-ATTO488) double-stranded probe corresponding to the consensus DNA binding sequence of c-REL (*c-REL* consensus probe) was prepared by annealing equimolar amounts of complementary DNA strands. Binding reactions were conducted with 100 nM purified c-REL in the presence or absence of 100 nM fluorescently labelled *c-REL* consensus probe in 10 mM Tris borate EDTA (pH 8.3), 10 mM NaCl, 40 mM KCl, 1 mM MgCl2, 1 mM DTT, and 0.2 mg/mL acetylated BSA at room temperature for 20 min. Competition assays were performed using various concentrations of unlabelled double-stranded DNA probes corresponding to putative c-REL binding sites or scrambled control sequences. The mobility of the protein–DNA complexes was analysed by non-denaturing 6% polyacrylamide gel electrophoresis at 100 V for 1 h. Fluorescent probes were detected using the Bio-Rad Gel Doc™ EZ System.

### 2.13 Liver histology

Formalin-fixed liver tissue was processed for haematoxylin-eosin staining and evaluated for lipid droplet content, microsteatosis, and macrosteatosis by bright-field microscopy.

### 2.14 Statistical analysis

Data are presented as mean values ± SEM. Statistical analyses were performed using GraphPad Prism software, with significance levels indicated as follows: *P < 0.05, **P < 0.01, ***P < 0.001, and ****P < 0.0001. The specific test used is detailed in the figure legend.

## 3. Results

### 3.1 Enrichment of c-REL at atherosusceptible sites by EC response to low shear stress

**Figure 1:**
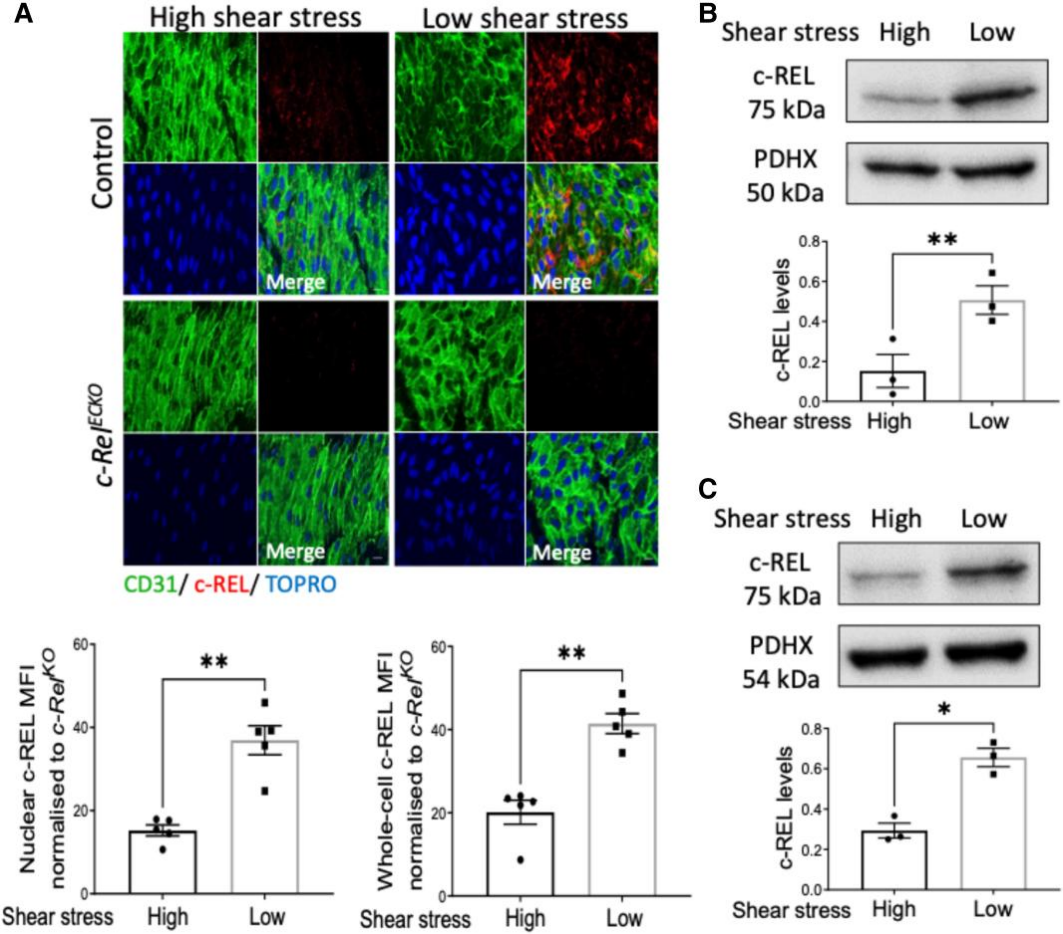
c-Rel is enriched at atheroprone sites via upregulation by low shear stress. (*A*) Aortic arches were isolated from control wild-type mice or from c*-Rel^KO^* mice, and *en face* immunostainings were performed using anti-c-REL antibodies. The endothelium was stained with anti-CD31 antibodies and co-stained with TOPRO-3 (DNA). Representative images are shown (scale bar = 10 μm). Each value in the graphs below represent the average nuclear (left) and whole-cell (right) c-REL mean fluorescence intensity (MFI) from 4 to 6 fields of view (10 cells quantified per field of view) from high or low shear stress regions of an individual control mouse (*n* = 5 mice) minus the MFI from c*-Rel^KO^* (*n* = 5 mice) mice. (*B* and *C*) Human ECs were exposed to low or high shear stress for 72 h using the orbital system. Levels of c-REL protein were analysed by immunoblotting with normalization to the level of PDHX in HUVEC (*B*) (*n* = 3 individual donors) or HCAEC (*C*) (*n* = 3 individual donors). Mean values are shown ±standard errors. Differences between means were analysed using a paired *t*-test. *P < 0.05 and **P < 0.01.

Analysis of publicly available single-cell RNAseq data revealed that c-Rel is expressed ubiquitously in multiple vascular cell types and immune cells in the murine aorta (see Supplementary material online, *Figure S1*).^27,28^ Focusing on endothelium, we investigated the potential correlation between shear stress and c-REL distribution by performing *en face* staining of murine aortic arch regions characterized by distinct shear stress profiles, as previously described.^29^ Strikingly, our analysis revealed heightened levels of both total c-REL and nuclear c-REL at the inner curvature of the aortic arch (low shear stress; atherosusceptible site) compared to the outer curvature (high shear stress; atheroprotected) (*Figure 1A*). To ensure staining specificity, we conducted parallel experiments in *c-Rel^KO^* mice, demonstrating the abolition of the fluorescent signal (*Figure 1A*). *In vitro* investigations further supported the relationship between c-REL and shear stress. We observed a significant elevation in c-REL protein expression in both HUVEC and HCAEC exposed to low shear stress compared to high shear stress conditions (*Figure 1B* and *C*). Collectively, our results indicate that c-REL is induced by low shear stress, leading to its enrichment at atherosusceptible regions within arteries.

### 3.2 c-REL co-ordinates endothelial inflammatory activation in response to low shear stress via TXNIP-p38 MAP kinase signalling

To elucidate the function of c-REL at atheroprone sites, we initially conducted bulk RNA analysis of HUVEC exposed to low shear stress and examined the impact of *c-REL* silencing on gene expression. Silencing of *c-REL* altered the expression of 2398 genes (1.2-fold; *P* < 0.05). Functional annotation using Metascape revealed multiple enriched Gene Ontology terms including those associated with inflammation (MAP kinase signalling, cellular response to cytokine stimulus, regulation of NF-κB signal transduction), cell proliferation (regulation of cell population proliferation, response to growth factor), and metabolism (protein catabolic process, mitochondrion organization, response to oxidative stress) (see Supplementary material online, *Figure S2A*).

**Figure 2:**
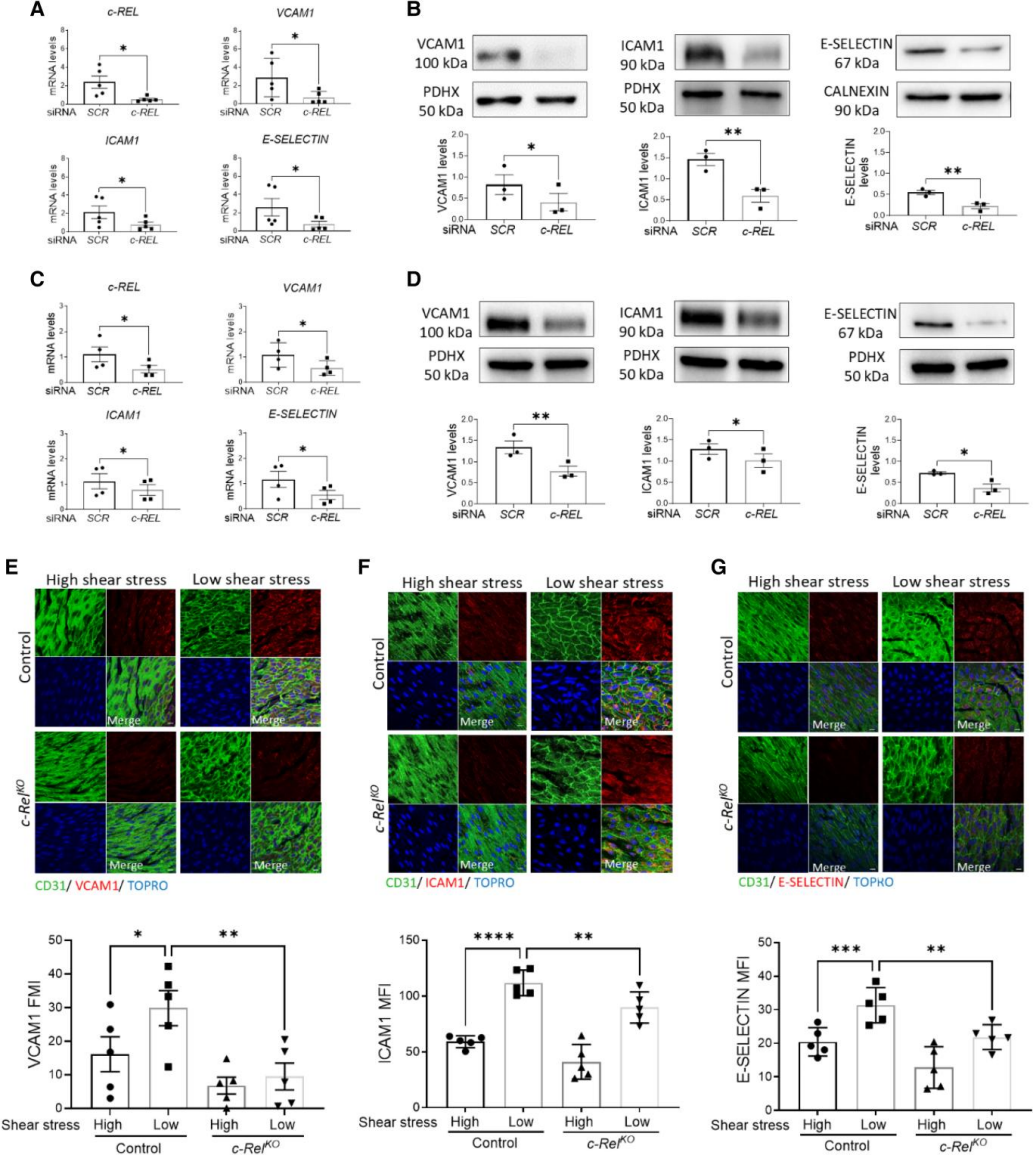
c-Rel promotes inflammation at atheroprone regions in response to low shear stress. (*A–D*) Human ECs were treated with *c-REL* siRNA or with scrambled non-targeting sequences (SCRs) and exposed to low shear stress for 72 h using the orbital system. Expression levels of *c-REL*, *VCAM1*, *ICAM1*, and *E-SELECTIN* were quantified by qRT-PCR in HUVEC (*A*) (*n* = 5 individual donors) and HCAEC (*C*) (*n* = 4 individual donors). Protein levels of VCAM1, ICAM1, and E-SELECTIN were analysed by immunoblotting and normalized to the level of PDHX in HUVEC (*B*) (*n* = 3 individual donors) and HCAEC (*D*) (*n* = 3 individual donors). (*E–G*) EC inflammatory activation was analysed at low or high shear stress regions of the aorta in control (*n* = 5 mice) vs. *c-Rel^KO^* (*n* = 5 mice) mice. *En face* immunostaining was performed using antibodies against VCAM1 (*E*), ICAM1 (*F*), or E-SELECTIN (*G*). ECs were identified using anti-CD31, and nuclei were co-stained with TOPRO-3. Representative images are shown (scale bar = 10 μm). Each value represents the average MFI from 5 to 6 fields of view (10 cells analysed per field of view) from high or low shear stress regions of an individual mouse. Mean values are shown ±standard errors. Differences between means were analysed using paired *t*-test (*A–D*) or two-way ANOVA (*E–G*). *P < 0.05, **P < 0.01, ***P < 0.001, and ****P < 0.0001.

We validated the potential link to inflammation by analysing the effects of *c-REL* silencing in cultured human EC exposed to low shear stress. It was observed that silencing of *c-REL* led to a significant reduction in the expression of the inflammatory adhesion molecules VCAM1, ICAM1, and E-SELECTIN in HUVECs at both mRNA and protein levels (*Figure 2A* and *B*). Similarly, silencing of *c-REL* led to reduction in VCAM1, ICAM1, and E-SELECTIN in HCAECs exposed to low (*Figure 2C* and *D*) or high shear stress (see Supplementary material online, *Figure S3A*). To further investigate the link between c-REL and inflammation, we recapitulated elevated c-REL in HCAEC exposed to low shear stress using a lentiviral system. This approach was validated by demonstrating enhanced levels of c-REL in cells transduced with lentiviral-c-REL compared to lentiviral control (see Supplementary material online, *Figure S4A*). Overexpression of c-REL induced elevated levels of VCAM-1, ICAM-1, and E-SELECTIN at both mRNA and protein levels (see Supplementary material online, *Figure S4B* and *C*), confirming the regulation of adhesion molecule expression by c-REL. Consistent with these *in vitro* results, *en face* staining of the murine aorta demonstrated heightened VCAM-1, ICAM-1, and E-SELECTIN expression at low shear stress regions compared to high shear stress regions (*Figure 2E–G*). Genetic deletion of *c-Rel* in mice significantly attenuated VCAM-1 (*Figure 2E*), ICAM-1 (*Figure 2F*), and E-SELECTIN (*Figure 2G*) expression at the low shear stress site, further supporting the role of c-REL as a positive regulator of inflammatory molecule expression.

**Figure 3:**
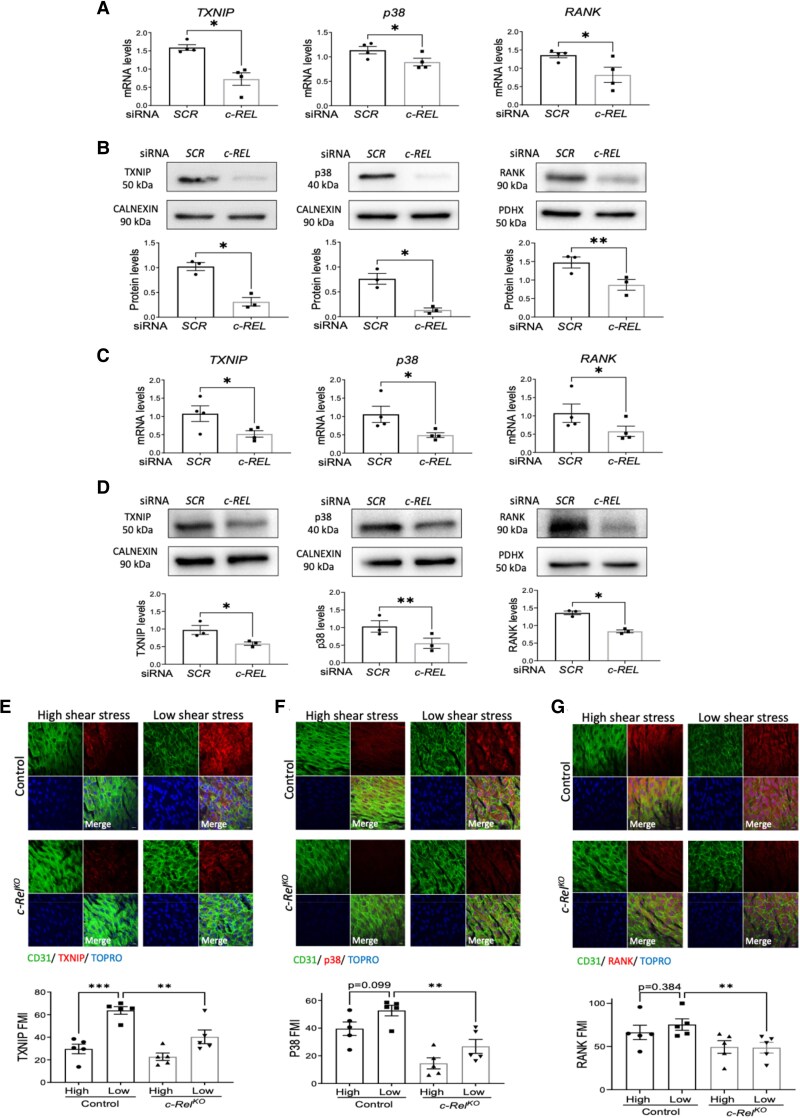
c-Rel positively regulates pro-inflammatory MAP kinase signalling at low shear stress regions. (*A–D*) Human ECs were treated with *c-REL* siRNA or with scrambled non-targeting sequences (SCR) and exposed to flow for 72 h using the orbital system. ECs were isolated from the centre (low shear stress) of wells prior to qRT-PCR (*A* and *C*) or immunoblotting (*B* and *D*). Expression levels of *TXNIP*, *p38*, and *RANK* were quantified by qRT-PCR in HUVEC (*A*) (*n* = 5 individual donors) and HCAEC (*C*) (*n* = 4 individual donors). Protein levels of TXNIP, P38, and RANK were analysed by immunoblotting and normalized to the level of CALNEXIN or PDHX in HUVEC (*B*) (*n* = 3 individual donors) and HCAEC (*D*) (*n* = 3 individual donors). (*E–G*) *En face* immunostaining was performed to quantify levels of TXNIP (*E*), P38 (*F*), and RANK (*G*) in EC at low or high shear stress regions of the aorta in control (*n* = 5 mice) vs. *c-Rel*^KO^ (*n* = 5 mice) mice. ECs were identified using anti-CD31, and nuclei were co-stained with TOPRO-3. Representative images are shown (scale bar = 10 μm). Each value represents the average MFI from 5 to 6 fields of view (10 cells analysed per field of view) in high or low shear stress regions of each mouse. Mean values are shown ±standard errors. Differences between means were analysed using paired *t-*tests (*A–D*) or two-way ANOVA (*E–G*). *P < 0.05, **P < 0.01, and ***P < 0.001.

Our unbiased transcript analysis pinpointed c-REL as a key regulator of multiple MAPK pathways (see Supplementary material online, *Figure S2B*), prompting us to hypothesize that MAPK regulation might underlie c-REL-driven endothelial activation. Consistent with this, silencing of *c-REL* led to a significant reduction in TXNIP, p38, and the TNF superfamily member RANK (*TNFRSK11A*) in HUVECs at both mRNA (*Figure 3A*) and protein (*Figure 3B*) levels. Similarly, silencing of c-REL led to reduction in TXNIP, P38, and RANK in HCAECs exposed to low (*Figure 3C* and *D*) or high (see Supplementary material online, *Figure S3B*) shear stress. On the contrary, overexpression of c-REL induced elevated levels of TXNIP, p38, and RANK at both mRNA and protein levels (see Supplementary material online, *Figure S4D* and *E*). Interestingly, overexpression of p38 or TXNIP did not rescue inflammatory molecule expression in cells where c-REL was silenced (see Supplementary material online, *Figure S5*). Our interpretation is that c-REL induces multiple signalling molecules each required for full inflammatory activation and that restoration of single members of this network is not sufficient to restore inflammation.

*In vitro* findings were substantiated by *en face* staining of the murine aortic arch demonstrated heightened endothelial expression of TXNIP, p38, and RANK at the low shear stress region, with genetic deletion of *c-Rel* leading to reduced expression of each of these proteins (*Figures 3E–G*). These data suggest that c-REL promotes inflammation at sites of low shear stress through augmentation of the p38 and its upstream regulators RANK and TXNIP.

To decipher the mechanism, we postulated that c-REL may induce an exogenous factor that drives inflammatory activation; however, transfer of cell culture medium from cultures where c-REL was silenced did not recapitulate the effects of c-REL silencing (see Supplementary material online, *Figure S6*). We next investigated whether c-REL binds directly to the promoters of adhesion molecules and inflammatory MAP kinase components. Bioinformatics analysis revealed potential c-REL binding sites near the transcriptional start sites of *VCAM-1*, *ICAM-1*, *E-selectin*, *P38*, *RANK*, and *TXNIP* (see Supplementary material online, *Figure S7*). Since high-quality antibodies for c-REL chromatin immunoprecipitation are unavailable, we analysed promoter binding empirically by electrophoretic mobility shift assay. The electrophoretic mobility of a *c-REL* consensus probe was retarded by purified c-REL (see Supplementary material online, *Figure S8*). This retardation was reduced in a dose-dependent manner by probes corresponding to putative c-REL binding sites within *VCAM-1*, *ICAM-1*, *E-SELECTIN*, *TXNIP*, *p38*, and *RANK* but was not altered by scrambled control sequences (see Supplementary material online, *Figure S8*), indicating interaction with each of the putative c-REL binding sequences. We conclude that c-REL is a driver of inflammatory activation in EC exposed to low shear stress conditions. The mechanism involves direct binding of c-REL to the promoters of genes encoding adhesion proteins and additionally involves the induction of pro-inflammatory MAP kinase signalling molecules.

### 3.3 c-REL promotes EC proliferation in response to low shear stress via a non-canonical NF-κB pathways

**Figure 4:**
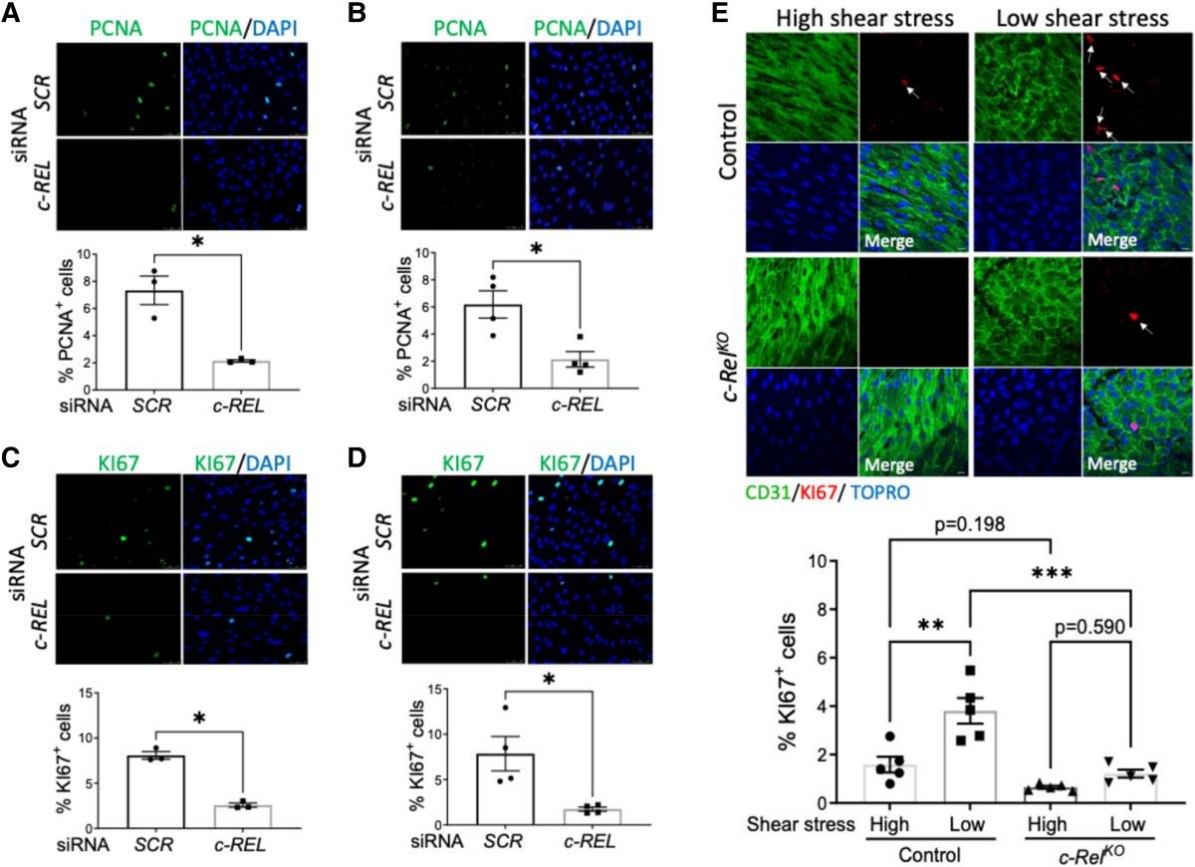
c-Rel promotes EC proliferation in response to low shear stress. (*A–D*) Human ECs were treated with *c-REL* siRNA or with scrambled non-targeting sequences (SCR) and exposed to low shear stress for 72 h using the orbital system. Proliferation was quantified by immunofluorescence staining using antibodies against PCNA (green) in HUVEC (*A*) (*n* = 3 individual donors) and in HCAEC (*B*) (*n* = 4 individual donors) and KI67 (green) in HUVEC (*C*) (*n* = 3 individual donors) and in HCAEC (*D*) (*n* = 3 individual donors). Each data point represents average values from 3 to 5 fields of view for each donor. Nuclei were co-stained with DAPI (blue) (scale bar = 50 μm). (*E*) EC proliferation was analysed at low or high shear stress regions of the aorta in control (*n* = 5 mice) vs. *c-Rel^KO^* (*n* = 5 mice) mice. *En face* immunostaining was performed using antibodies against Ki67. ECs were identified using anti-CD31, and nuclei were co-stained with TOPRO-3. Representative images are shown (scale bar = 10 μm). The proportion of Ki67-positive cells was averaged from 5 to 7 fields of view in of high or low shear stress regions of each mouse. Mean values are shown ±standard errors. Differences between means were analysed using paired *t*-tests (*A* and *D*) or two-way ANOVA (*E*). *P < 0.05, **P < 0.01, and ***P < 0.001.

We next explored the role of c-REL in EC proliferation. Gene silencing of *c-REL* resulted in a significant reduction in the percentage of proliferating HUVEC or HCAEC exposed to low shear stress, as indicated by the proportion of cells that expressed the markers PCNA (*Figure 4A* and *B*) and Ki67 (*Figure 4C* and *D*). Conversely, the impact of *c-REL* silencing in EC exposed to high shear stress was relatively modest (see Supplementary material online, *Figure S9*). We obtained further evidence by overexpression of c-REL in HCAEC under low shear stress, which significantly elevated the proportion of cells that expressed PCNA and Ki67 (see Supplementary material online, *Figure S10*). *En face* staining of Ki67 in the murine aorta consistently revealed enhanced EC proliferation in low shear stress conditions compared to high shear stress conditions (*Figure 4E*). Furthermore, genetic deletion of *c-Rel* significantly attenuated proliferation at low shear stress sites in the aorta (*Figure 4E*) suggesting that *c-Rel* contributes to augmented focal EC proliferation at sites of low shear stress.

To decipher the mechanism, we initially analysed whether c-REL influences other members of the NF-κB family, which exhibit considerable cross-talk and play a pivotal role in proliferation.^30^ qRT-PCR revealed that silencing of *c-REL* reduced mRNA levels of *NFKB2* but did not influence *RELB*, *NFKB1*, *RELA*, or the upstream NF-κB signalling molecules *CHUK*, *IKBKB*, *IKBKG*, or *NFKB1A* (see Supplementary material online, *Figure S11A*). Similarly, immunofluorescent staining demonstrated that c-REL silencing led to a reduction in total and nuclear p52 levels but did not alter other NF-κB family members (see Supplementary material online, *Figure S11B–E*).

**Figure 5:**
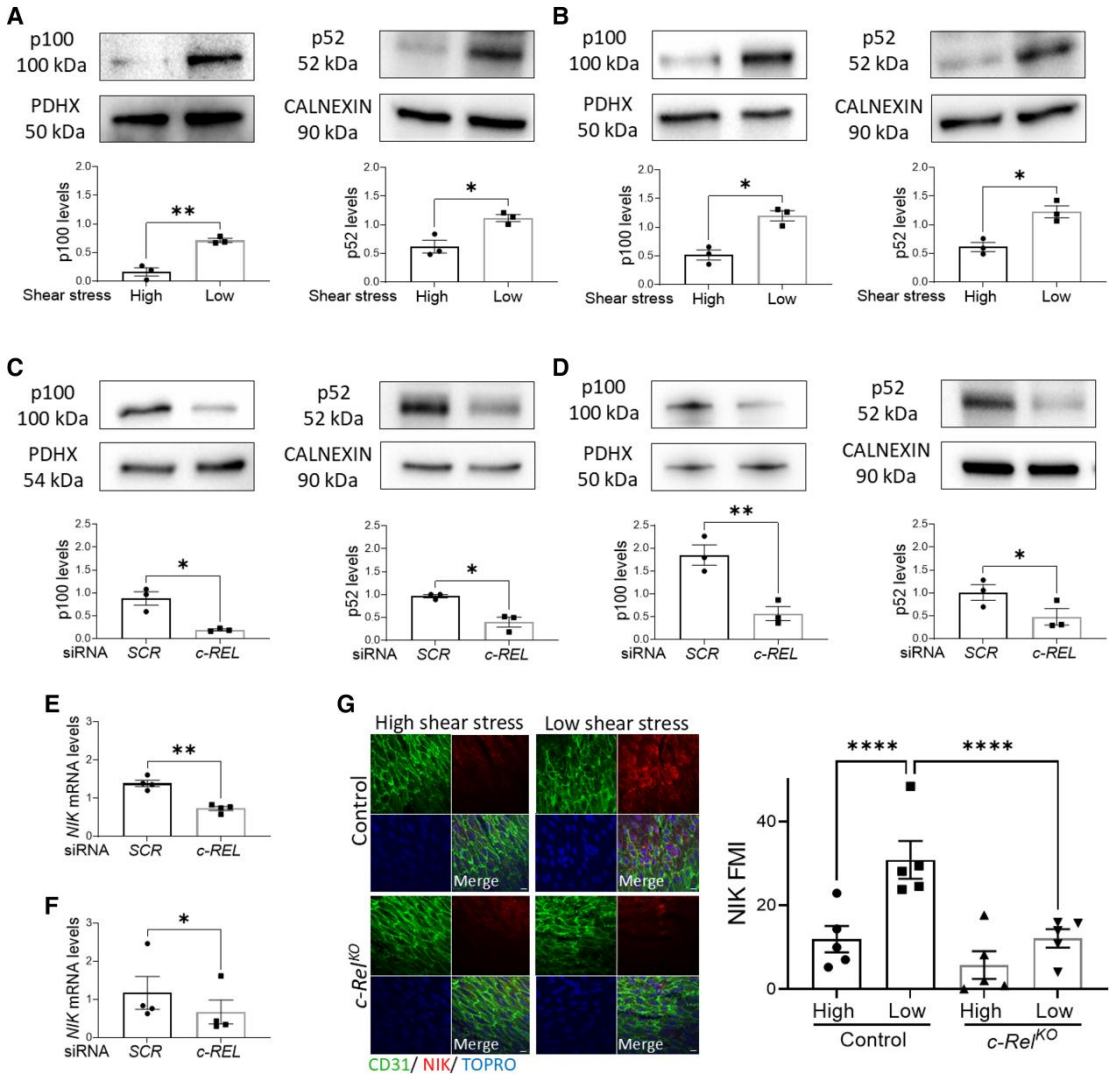
c-Rel enhances non-canonical NFKB2 activation in response to low shear stress. (*A* and *B*) Human ECs were exposed to low or high shear stress for 72 h using the orbital system. (*C* and *D*) Human ECs were treated with *c-REL* siRNA or with scrambled non-targeting sequences (SCR) and exposed to low shear stress for 72 h. (*A–D*) Levels of the *NFKB2* gene products P100 and P52 were analysed by immunoblotting with normalization to the level of PDHX in HUVEC (*A* and *C*) (*n* = 3 individual donors) and HCAEC (*B* and *D*) (*n* = 3 individual donors). (*E* and *F*) Human ECs were treated with *c-REL* siRNA or with SCR and exposed to low shear stress for 72 h. Expression levels of *NIK* were quantified by qRT-PCR in HUVEC (*E*) (*n* = 4 individual donors) and HCAEC (*F*) (*n* = 4 individual donors). (*G*) *En face* immunostaining was performed to quantify levels of NIK in EC at low or high shear stress regions of the aorta in control (*n* = 5 mice) vs. *c-Rel^KO^* (*n* = 5 mice) mice. ECs were identified using anti-CD31, and nuclei were co-stained with TOPRO-3. Representative images are shown (scale bar = 10 μm). Each value represents the average MFI from 5 to 6 fields of view (10 cells quantified per field of view) in high or low shear stress regions in each mouse. Mean values are shown ±standard errors. Differences between means were analysed using paired *t*-tests (*A–F*) or using two-way ANOVA (*G*). *P < 0.05, **P < 0.01, and ****P < 0.0001.

We next focused on the non-canonical NF-κB signalling pathway which acts upstream from *NFKB2*. This pathway is initiated through the activation of NF-κB-inducing kinase NIK (*MAP3K14*), which facilitates the proteolytic processing of p100 into p52, subsequently translocating to the nucleus. Immunoblotting revealed that low shear stress heightened the expression of p100 and the active p52 NF-κB subunit in HUVEC (*Figure 5A*) and HCAEC (*Figure 5B*). Moreover, *c-REL* silencing significantly attenuated the expression of both p100 and p52 in HUVEC (*Figure 5C*) and HCAEC (*Figure 5D*). Upstream from p100/p52, the expression of *NIK* was reduced by silencing of *c-REL* in HUVEC (*Figure 5E*) and HCAEC (*Figure 5F*). We concluded that *c-REL* can interact with a putative *c-REL* binding site within the promoter of *NIK* since a probe corresponding to this region competed for c-REL binding in an electrophoretic mobility shift assay (see Supplementary material online, *Figure S8*). Consistently, *en face* staining of the murine aorta revealed enhanced expression of NIK at the low shear region and analysis of *c-Rel^KO^* mice demonstrated that *c-Rel* was necessary for NIK enrichment at this site (*Figure 5G*).

We deduced that non-canonical NF-κB serves as a driver of EC proliferation because genetic deletion of *NFKB2* exhibited a trend towards reduced proliferation at low shear stress sites in the aorta (see Supplementary material online, *Figure S12A*). Similarly silencing of *NFKB2* notably diminished the percentage of proliferating cells in HUVEC and HCAEC exposed to low shear stress conditions *in vitro* (see Supplementary material online, *Figure S12B* and *C*). At the mechanistic level, the expression of the cell cycle inhibitor p21 (*CDKN1A*) was enhanced by silencing of *NFKB2* (see Supplementary material online, *Figure S12D–G*) or *c-REL* (see Supplementary material online, *Figure S12H* and *I*) in HUVEC or HCAEC exposed to low shear stress. Collectively, these findings suggest that c-REL drives EC proliferation at sites of low shear stress by activating non-canonical NF-κB signalling to repress p21. Consistent with the role of p21 in senescence, we observed that *c-REL* silencing enhanced the expression of two markers of senescence, H2AX and p53, in HCAEC exposed to low shear stress (see Supplementary material online, *Figure S13A* and *B*). By contrast, *c-REL* silencing did not alter apoptosis in HCAEC and had relatively modest effects in HUVEC (see Supplementary material online, *Figure S13C* and *D*).

### 3.4 Endothelial c-Rel promotes atherosclerosis

We hypothesized that c-REL may influence atherosclerosis given its pivotal role in regulating vascular inflammation. We initially analysed mice with whole-body *c-Rel* deletion (*c-Rel^KO^*) which are viable and have a seemingly normal development, although they show a deficiency in lymphocyte proliferation and activation.^31^ Administration of AAV-PCSK9 and exposure to a western diet for 6 weeks revealed a reduction in aortic atherosclerotic plaques in *c-Rel^KO^* mice compared to controls (see Supplementary material online, *Figure S14A*). Interestingly, plasma triglycerides and cholesterol levels were reduced in *c-Rel^KO^* mice compared to controls (see Supplementary material online, *Figure S14B*). To validate this result, we next assessed the influence of IT603, a pharmacological inhibitor of c-REL, on plasma lipid levels in hypercholesterolaemic mice. Mice were treated with AAV-PCSK9 and western diet for 6 weeks with concurrent treatment with IT603 from Weeks 3 to 6. Treatment with IT603 did not influence plaque size under this regime but significantly reduced plasma levels of LDL cholesterol (see Supplementary material online, *Figure S15*). To begin to understand the mechanism linking *c-Rel* to plasma LDL cholesterol, we analysed whether *c-Rel^KO^* mice exhibited alterations in liver steatosis or lipid regulators in hypercholesterolaemic mice. The degree of steatosis and frequency of lipid droplets in the liver was similar in *c-Rel^KO^* mice and controls (see Supplementary material online, *Figure S14C*). qRT-PCR was used to determine expression levels of multiple regulators of the cholesterol (*Srebp2*, *Cyp51*, *Cyp7a1*, *Abca1*) and triglyceride (*Srebp1*, *Dgat2*, *Elov5*, *Elov6*, *Fabp1*, *Cd36*) pathways. Livers from *c-Rel^KO^* mice exhibited reduced expression levels of *Abca1*, *Srebp2*, and *Elov6* with a trend towards statistical significance (see Supplementary material online, *Figure S14D*). We conclude that *c-Rel* regulates plasma cholesterol levels, possibly involving hepatic cholesterol metabolism.

**Figure 6:**
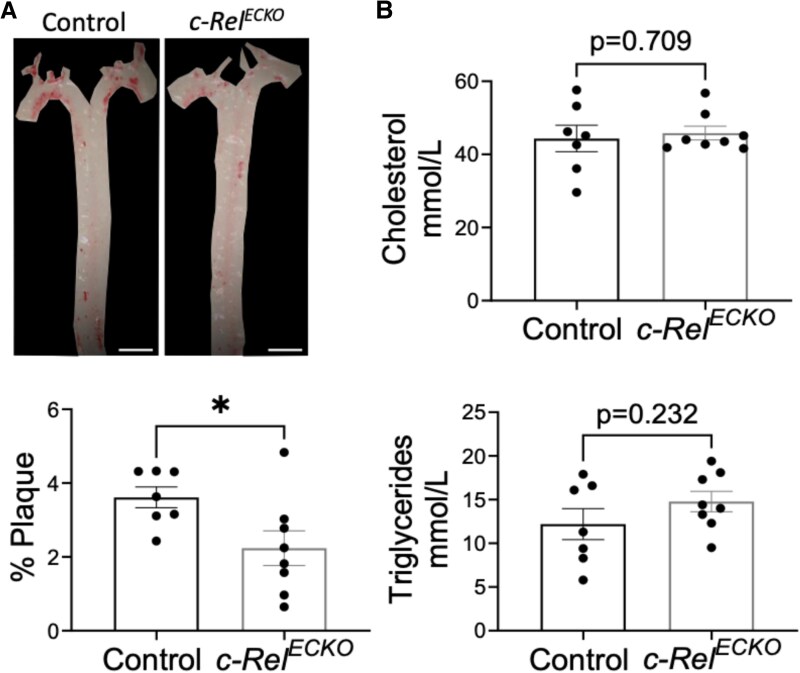
Endothelial c-Rel drives atherosclerosis. (*A* and *B*) *c-Rel^ECKO^* mice (*n* = 8 mice) aged 9 weeks and littermates lacking *Cre* (controls; *n* = 7 mice) received five IP injections of tamoxifen and one injection of PCSK9-AAV virus 1 week later. Mice were fed with western for 6 weeks. (*A*) Representative images of aortas stained with Oil Red O (upper) and quantification of plaque burden by calculating the percentage of aortic surface area covered by plaque (lower) (scale bar = 2 mm). (*B*) Total plasma cholesterol and triglyceride levels were measured and mean levels±standard errors are shown. Differences between means were analysed using an unpaired *t*-test. *P < 0.05, **P < 0.01, and ***P < 0.001.

Analysis of global c-REL knockout mice does not delineate the function of c-REL in EC vs. other cell types. Therefore, to specifically dissect the influence of endothelial c-REL on atherosclerosis, we selectively deleted *c-Rel* from the endothelium using the *Cdh5^CreERT2/+^* line crossed with a *c-Rel^fl/fl^* mouse, generating *c-Rel^ECKO^* and control mice. Following AAV-PCSK9 treatment and exposure to a western diet for 8 weeks to induce hypercholesterolaemia, *c-Rel^ECKO^* mice exhibited significantly smaller aortic atherosclerotic plaques compared to controls (*Figure 6A*), while plasma cholesterol and triglyceride levels remained unchanged (*Figure 6B*). These findings underscore endothelial *c-Rel* as a driver of atherosclerosis which is consistent with its ability to promote inflammation and endothelial turnover at atheroprone regions of the arterial tree.

Collectively, these findings indicate that endothelial *c-Rel* promotes atherosclerosis by inducing pro-atherogenic inflammatory molecules at disease-prone regions. Moreover, targeting c-REL may offer a dual benefit of protecting the vasculature and lowering plasma lipoprotein levels to attenuate atherosclerosis.

## 4. Discussion

The canonical NF-κB pathway controls multiple and diverse functions in many different tissues and cells. c-REL is traditionally known for its role in lymphocyte development,^32^ but also plays a part in various pathologies, including inflammatory and autoimmune diseases,^33^ fibrosis,^20,34,35^ and cancer immunosurveillance.^36^ Previous studies detected c-REL subunits in ECs and other cell types within human atherosclerotic plaques.^9^ Despite this, the potential involvement of c-REL in atherosclerosis pathology has remained unexplored.

Here, we demonstrate that c-REL exhibits increased expression and nuclear localization in EC at sites of low shear stress which are predilection sites for atherogenesis. The mechanoreceptors and associated signalling pathways that enhance c-REL at sites of low shear stress are unknown. Several mechanosensitive pathways including PI3 K, PKCζ, and TGF-β signalling regulate post-translational modifications of c-REL in other contexts, and their potential role in c-REL activation at sites of low shear stress should be analysed in future studies.^37,38^

Our research demonstrates that endothelial c-REL is a key regulator of inflammation and EC proliferation at sites of low shear stress. Inflammation is a pivotal factor in driving atherosclerosis, but the relationship between endothelial proliferation and atherosclerosis is intricate. EC proliferation is essential for vascular homeostasis; therefore, molecules suppressing basal proliferation have proatherogenic effects.^23^ However, excessive EC proliferation can induce vascular leakiness as mitotic cells round up,^7^ creating hot spots for lipoprotein influx from the circulation to the vessel wall, thereby promoting atherosclerosis. Notably, we found that genetic deletion of *c-Rel* in ECs results in a substantial reduction in inflammatory molecule expression and proliferation at sites of low shear stress coupled to reduced atherogenesis. This underscores that inflammation and proliferation driven by endothelial c-REL contribute to early atherosclerosis.

Our investigation of the mechanism of c-REL-driven inflammation involved an unbiased bulk RNA analysis where one of the most enriched Gene Ontology terms identified was MAP kinase signalling. Significantly, c-REL was found to regulate p38 and its upstream regulators RANK and TXNIP. This observation was substantiated through qPCR and immunoblotting in cultured cells using gene silencing and overexpression approaches and further confirmed *in vivo* by demonstrating reduced levels of p38, RANK, and TXNIP following *c-Rel* genetic deletion. Moreover, c-REL interacted with sequences corresponding to the promoters of these genes suggesting a potential transcriptional mechanism. Our findings align with the Berk lab, revealing TXNIP enrichment under disturbed flow and its pivotal role in p38 activation, crucial for adhesion protein expression and monocyte recruitment.^5,39^ Additionally, we discovered that c-REL induces RANK, acting upstream of both the TXNIP-p38 and non-canonical NF-κB pathways.^40,41^ While RANK is conventionally associated with bone physiology, its induction in angiogenic ECs suggests a vascular role.^42^ Genetic studies linking RANK allelic variation to coronary artery disease in inflammatory vasculitis further support this.^43^ Our data position c-REL as a novel player upstream in the RANK-TXNIP-p38 pathway, driving inflammation at atheroprone sites.

To define the mechanism linking c-REL to EC proliferation, we initially investigated crosstalk with other NF-κB family members since these drive proliferation in cancer cells and other contexts. We observed that c-REL specifically promotes non-canonical NF-κB2 activation by increasing the expression of p100, the precursor of the p52 NF-κB2 subunit, and by enhancing the expression of RANK and NIK which signal to promote p100 to p52 processing. The NIK-NF-κB2 pathway is recognized for driving cell proliferation in cancer by suppressing the expression of the cell cycle inhibitor p21.^44^ We investigated whether this pathway is conserved in EC and demonstrated that c-REL-NFKB2 signalling represses p21 under low shear stress conditions. P21 is a central regulator of transient and irreversible forms of growth arrest such as senescence.^45^ Intriguingly, c-REL suppressed markers of senescence in EC exposed to low shear stress. This suggests that c-REL may promote EC proliferation at sites of low shear stress, in part, by preventing irreversible growth arrest. Our observations do not rule out additional mechanisms linking c-REL to inflammatory activation or EC proliferation. Indeed, our unbiased bulk RNA analysis revealed c-REL regulation of metabolic and catabolic pathways and oxidative stress pathways that have clear links to inflammation and cell turnover.^46,47^ Further work is needed to determine whether these pathways contribute to c-REL-driven EC phenotypic change and early atherogenesis.

Our group and others have previously shown that RELA contributes to arterial inflammation is sites of low shear stress.^11–14^ However, the function of RELA extends beyond inflammation through the induction of anti-apoptotic molecules that are essential for vascular viability.^15,16^ This is apparent in mice with constitutive deletion of *RelA* which do not survive into adulthood due to the development of vascular damage associated with apoptosis.^17^ In contrast, mice lacking *c-Rel* are viable^20^ despite defects in myeloid and lymphoid immunity^18,31,32,48^ that could potentially influence atherosclerosis. We explored the effects of global suppression of c-REL on atherosclerosis using two approaches: constitutive *c-Rel* deletion and pharmacological inhibition using IT603. Our analysis of constitutive *c-Rel^KO^* mice confirmed an essential role in atherosclerosis, with noticeably smaller plaques. Intriguingly, constitutive *c-Rel* deletion and treatment with IT603 led to reduced plasma levels of plasma LDL cholesterol. *c-Rel^KO^* mice also exhibited a trend towards altered expression of *Srebp2* and *Abca1*, which is consistent with previous research implicating c-REL in the control of liver metabolic pathways.^49,50^ Further research is needed to clarify the mechanism linking c-REL to the regulation of plasma LDL cholesterol levels.

In summary, we show that endothelial c-REL orchestrates the initiation of atherosclerosis at sites of disturbed flow by activating a TXNIP-p38 pathway leading to inflammation and a NF-κB2-p21 pathway driving proliferation. Inhibition of c-REL provides a novel therapeutic strategy to enhance EC function and reduce atherosclerosis.

## Supplementary Material

cvaf024_Supplementary_Data

## Data Availability

The data underlying this article are available in the article and in its online supplementary material.
