# Supplementary material for: Endothelial c-REL orchestrates atherosclerosis at regions of disturbed flow through crosstalk with TXNIP-p38 and non-canonical NF-κB pathways
Source: Cardiovasc Res. 2025 Feb 21;121(5):748–59. doi: 10.1093/cvr/cvaf024 (PMC12101352; doi:10.1093/cvr/cvaf024)
Supplement: cvaf024_Supplementary_Data [file cvaf024_supplementary_data.docx]

**SUPPLEMENTAL MATERIAL**

**Endothelial c-REL orchestrates atherosclerosis at regions of disturbed flow through crosstalk with TXNIP-p38 and non-canonical NF-κB pathways.**

Blanca Tardajos Ayllon^1,2^, Neil Bowden^2^, Celine Souilhol^2^, Hazem Darwish^2^, Siyu Tian^1,2^, Carrie Duckworth^3^, D. Mark Pritchard^3^, Suowen Xu^4^, Jon Sayers^2^, Sheila Francis^2^, Jovana Serbanovic-Canic^2^, Fiona Oakley^5^, Paul C Evans^1^

**
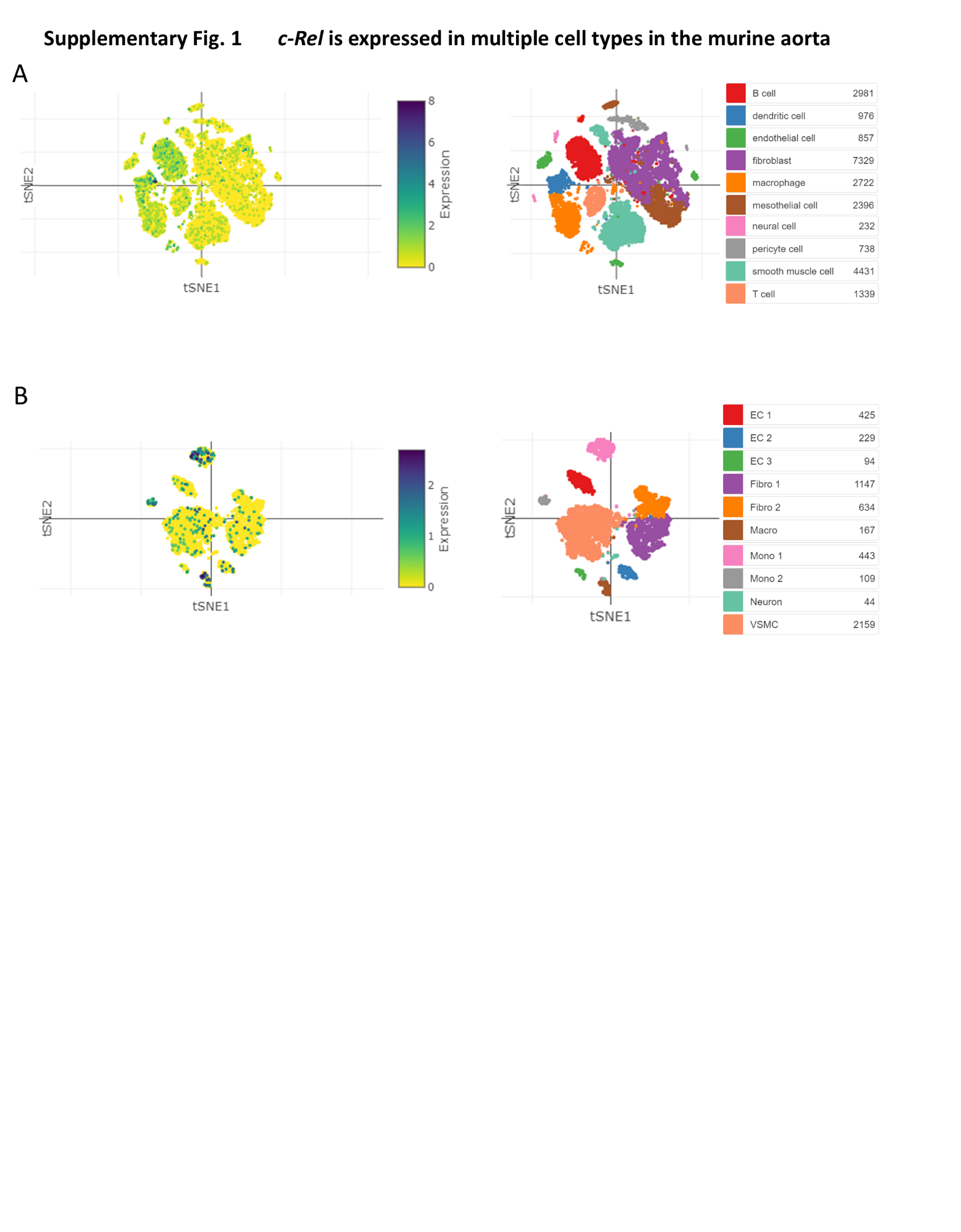
**

**Supplementary Fig. 1: *c-Rel* is expressed in multiple cell types in the murine aorta.**

Single Cell Portal Software was used to visualise c-Rel expression in publicly available scRNAseq datasets of murine aortas. (**A**) t-SNE representation of the scRNAseq data from Kan et al.^1^ showing *c-Rel* expression (left) in different cell type clusters (right). **(B)** t-SNE representation of the scRNAseq data from Kalluri et al.^2^ showing *c-Rel* expression (left) in different cell type clusters (right)**.**

**
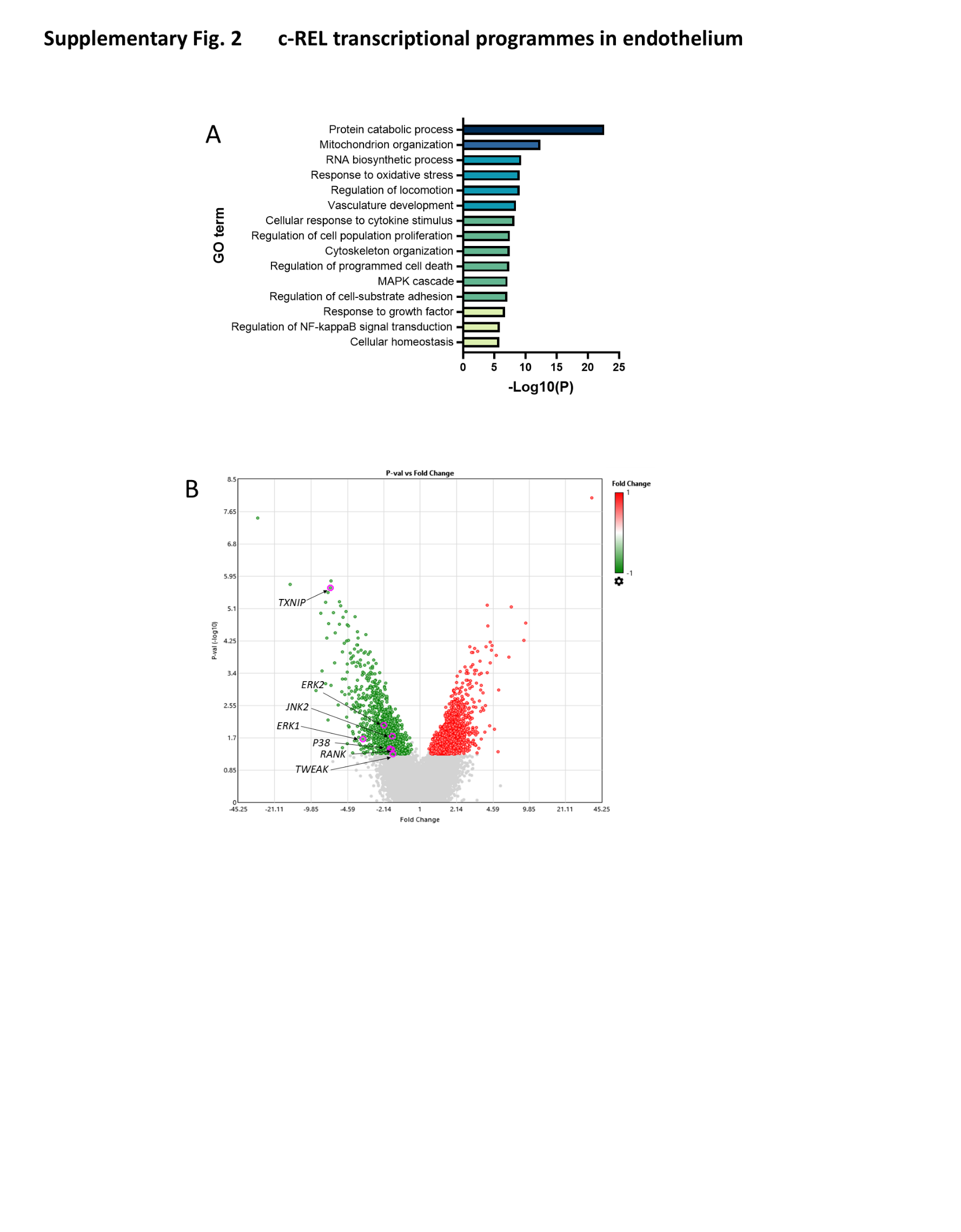
**

**Supplementary Fig. 2: c-REL transcriptional programs in endothelium.**

HUVEC were treated with *c-REL* siRNA or with scrambled non-targeting sequences and exposed to low shear stress for 72h using the orbital system (n=3 individual donors). **(A)** Bulk RNA analysis was coupled to functional enrichment of genes positively regulated by *c-REL* using Metascape software. Enrichment scores are represented as − log10 (p-value). (**B**) Bulk RNA analysis represented as a volcano plot displaying differentially expressed genes between *c-REL*-silenced and control samples. Green dots represent *c-REL*-positively regulated genes, red dots represent *c-REL*-negatively regulated genes, and gray dots represent genes that were not differentially expressed (P<0.05, fold change>1.2). Differentially expressed genes involved in MAP kinase signalling are highlighted in pink.

**Supplementary Fig. 3: c-Rel promotes inflammatory markers in response to high shear stress.** HCAECs were treated with *c-REL* siRNA or with scrambled non-targeting sequences (SCR) and exposed to high shear stress for 72h using the orbital system. Expression levels of **(A)** *c-REL*, *VCAM1*, *ICAM1* and *E-SELECTIN* and **(B)** *TXNIP, p38* and *RANK* were quantified by qRT-PCR (n=4 individual donors). Mean values are shown +/- standard errors. Differences between means were analysed using a paired *t-*test**.** *p<0.05, **p< 0.01.

**
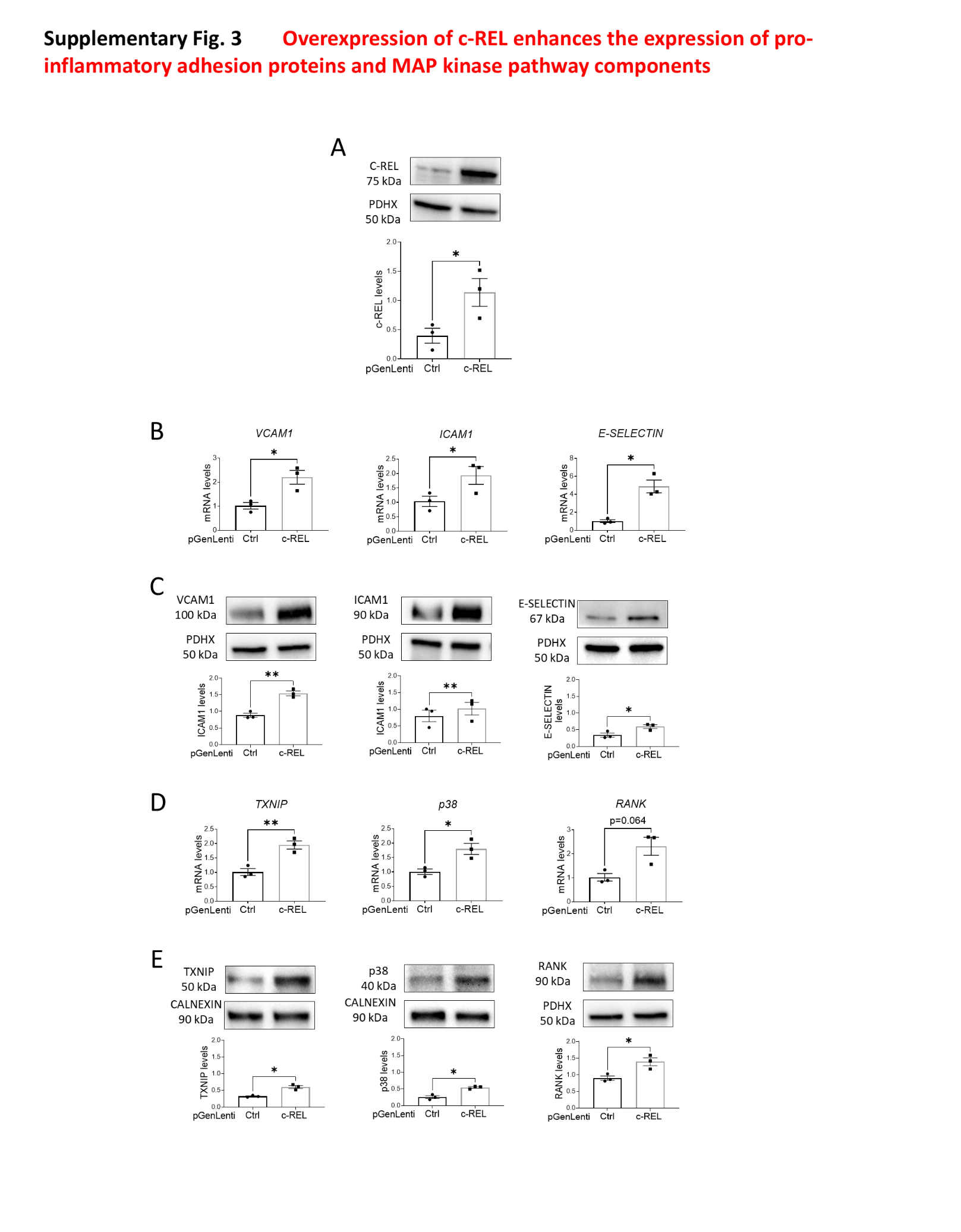
**

**Supplementary Fig. 4: Overexpression of c-REL enhances the expression of pro-inflammatory adhesion proteins and MAP kinase pathway components. (A-E)** HCAECs were transfected with c-REL or control pGenLenti lentiviral vectors and exposed to flow for 72h using the orbital system. EC were isolated from the centre (low shear stress) of wells prior to immunoblotting **(A, C, E)** or qRT-PCR **(B, D)**. **(A, C, E)** Protein levels of c-REL, VCAM1, ICAM1, E-SELECTIN, TXNIP, P38 and RANK were analysed by immunoblotting and normalised to the level of CALNEXIN or PDHX (n=3 individual donors). (**B, D**) Expression levels of *VCAM1, ICAM1, E-SELECTIN,* *TXNIP*, *p38* and *RANK* were quantified by qRT-PCR (n=3 individual donors). Mean values are shown +/- standard errors. Differences between means were analysed using a paired t-test. *P<0.05, **P<0.01.


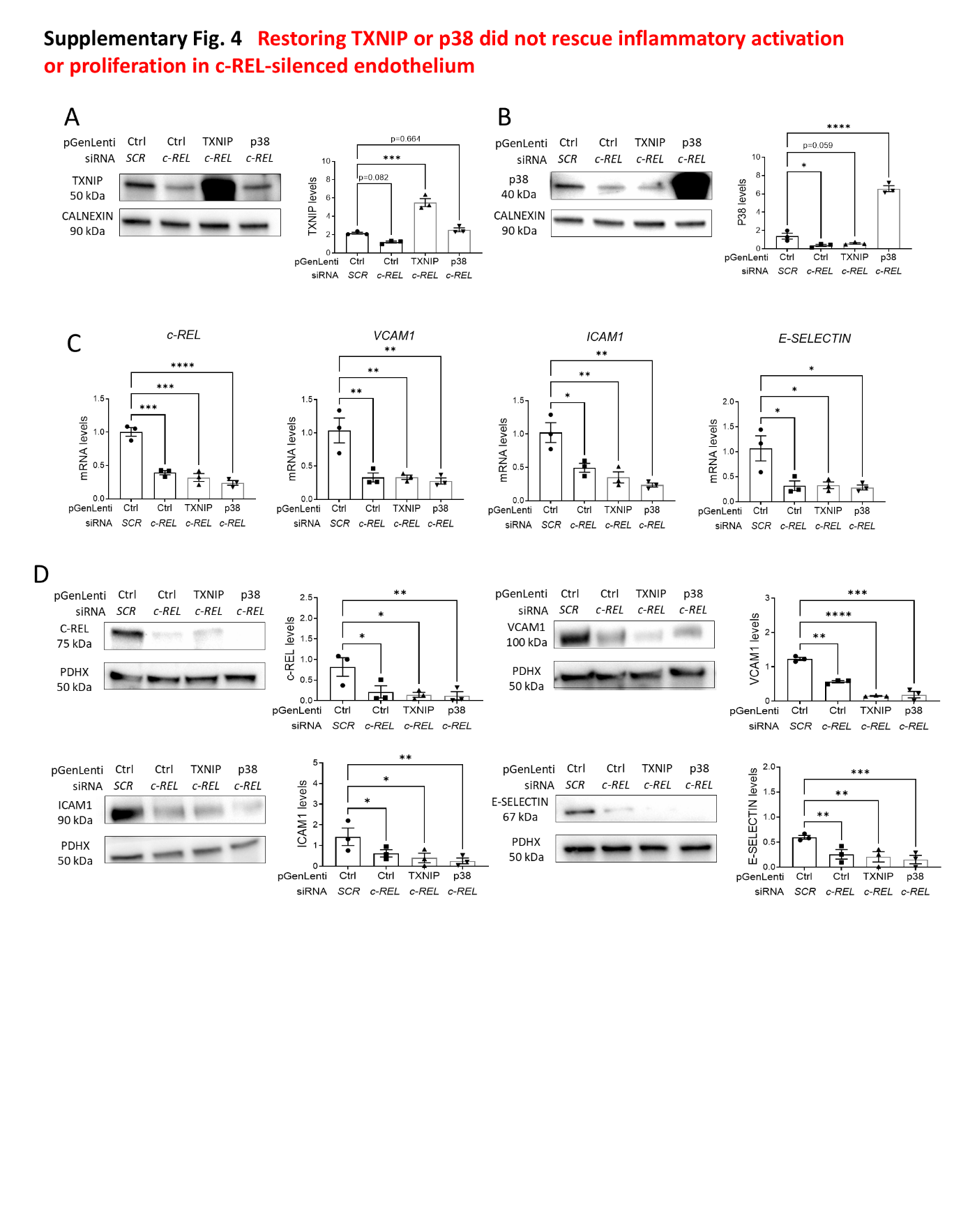


**Supplementary Fig. 5: Restoring TXNIP or p38 did not rescue inflammatory activation or proliferation in c-REL-silenced endothelium.**

**(A-D)** HCAECs were treated with *c-REL* siRNA or with scrambled non-targeting sequences (SCR) and they were then transfected with TXNIP, p38 or control pGenLenti lentiviral vectors. **(A, B, D)** Protein levels of TXNIP, p38, c-REL, VCAM1, ICAM1 and E-SELECTIN were analysed by immunoblotting and normalised to the level of CALNEXIN or PDHX (n=3 individual donors). (**C**) Expression levels of *c-REL*, *VCAM1, ICAM1* and *E-SELECTIN* were quantified by qRT-PCR (n=3 individual donors). Mean values are shown +/- standard errors. Differences between means were analysed using a 2-way ANOVA. *P<0.05, **P<0.01, ***P<0.001, ****P<0.0001.

**
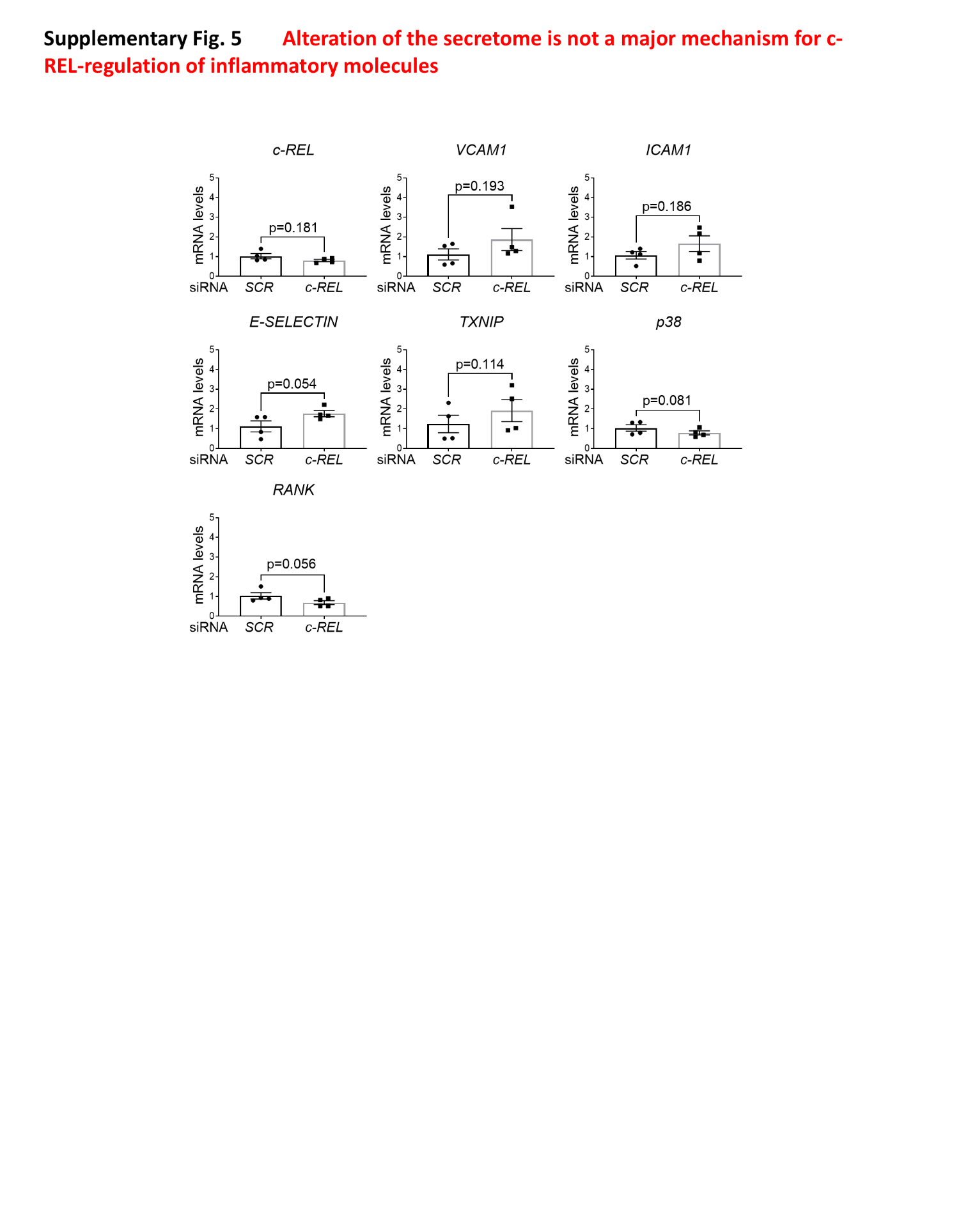
**

**Supplementary Fig. 6: Alteration of the secretome is not a major mechanism for c-REL-regulation of inflammatory molecules.**

HCAECs were treated with scrambled non-targeting sequences (SCR) and cell culture medium from different HCAEC treated with *c-REL* siRNA was transferred to the cells. Cells were exposed to low shear stress for 72h using the orbital shaker. Expression levels of *c-REL*, *VCAM1, ICAM1, E-SELECTIN, TXNIP*, *p38* and *RANK* were quantified by qRT-PCR (n=4 individual donors). Mean values are shown +/- standard errors. Differences between means were analysed using a paired *t-*test.

**
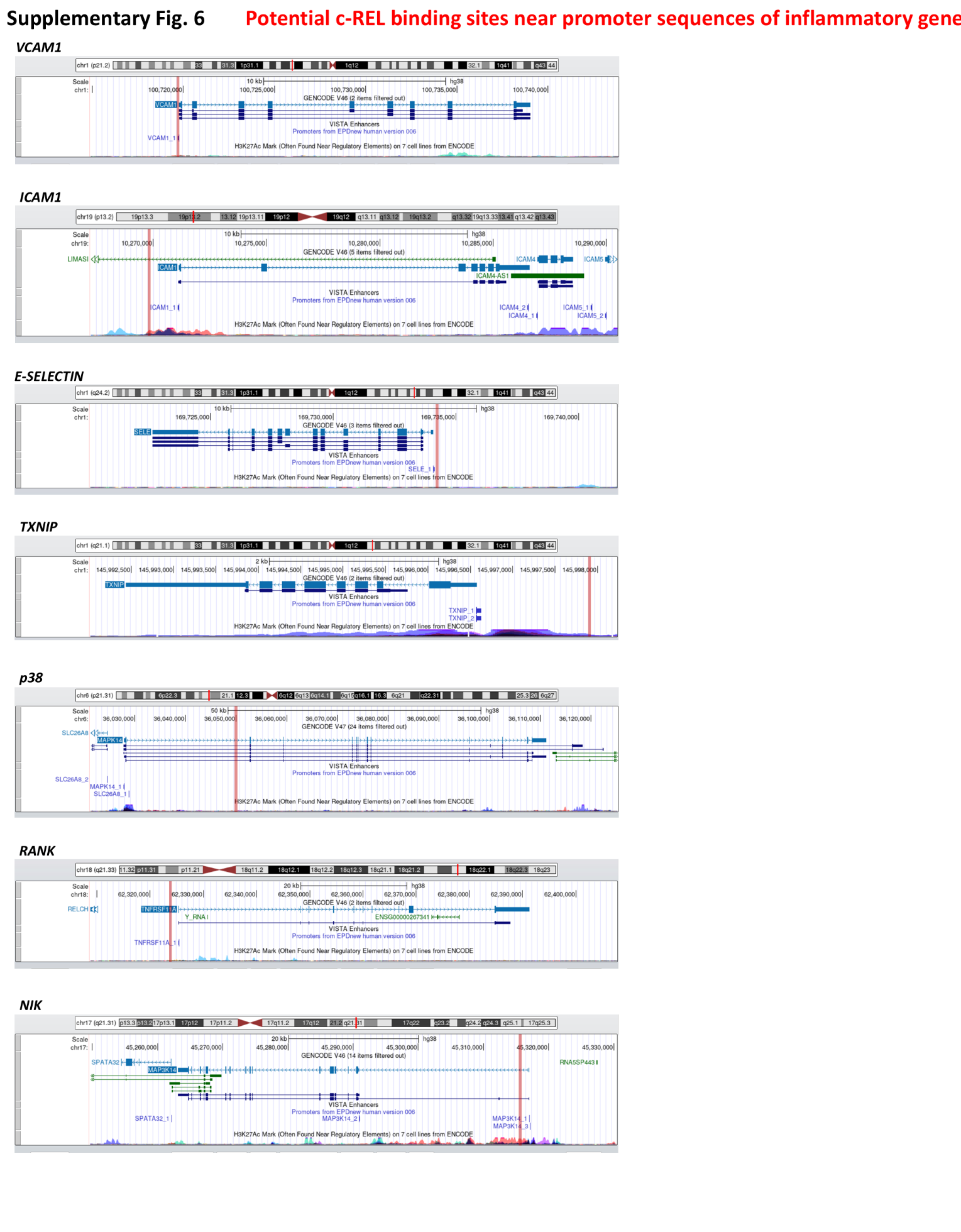
**

**Supplementary Fig. 7: Potential *c-REL* binding sites near promoter sequences of inflammatory genes.**

Bioinformatic analysis of potential *c-REL* binding sites near the promoter sequences of *VCAM1, ICAM1, E-SELECTIN, TXNIP*, *p38,* *RANK* and *NIK* was performed using the UCSC Genome Browser website. Vertical red lines are shown to indicate *c-REL* potential binding sites.

**
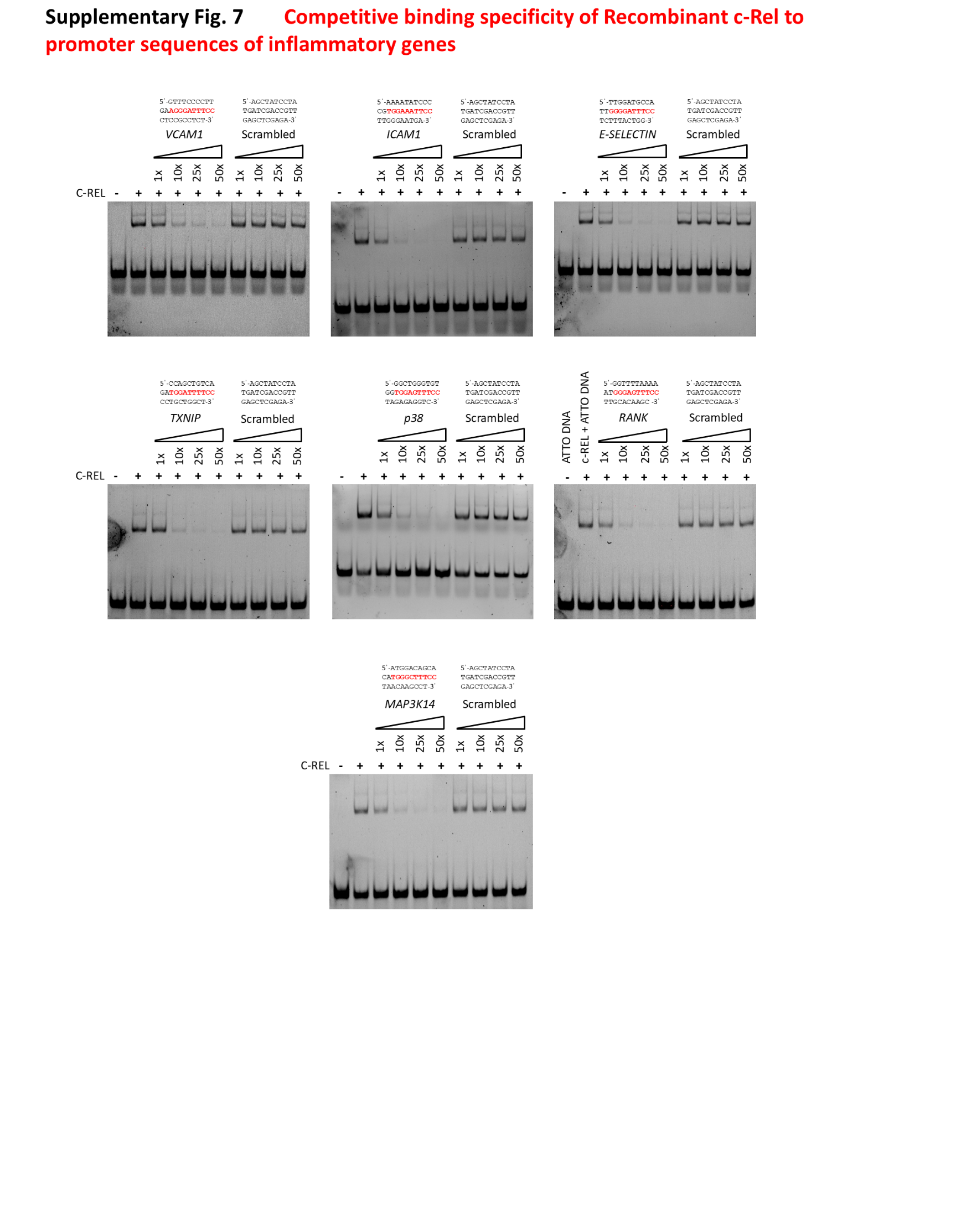
**

**Supplementary Fig. 8: Probes representing putative *c-REL* binding sites from *VCAM-1, ICAM-1, E-SELECTIN, TXNIP, p38* and *RANK* genes interact with purified c-REL.** Fluorescent *c-REL* consensus probe was incubated in the absence (-) or presence (+) of equimolar concentrations of purified c-REL. Reactions were carried out in the presence of varying concentrations (from equimolar 1X to 50X molarity) of unlabelled double stranded DNA competitors that represent promoter regions of *VCAM1, ICAM1, E-SELECTIN, TXNIP*, *p38,* *RANK* and *NIK* containing putative *c-REL* binding sites (red font). Alternatively, reactions were carried out in the presence of varying concentrations of scrambled control sequences. EMSA was carried out and probe (lower bands) and probe/c-REL complexes (upper bands) were identified.

**
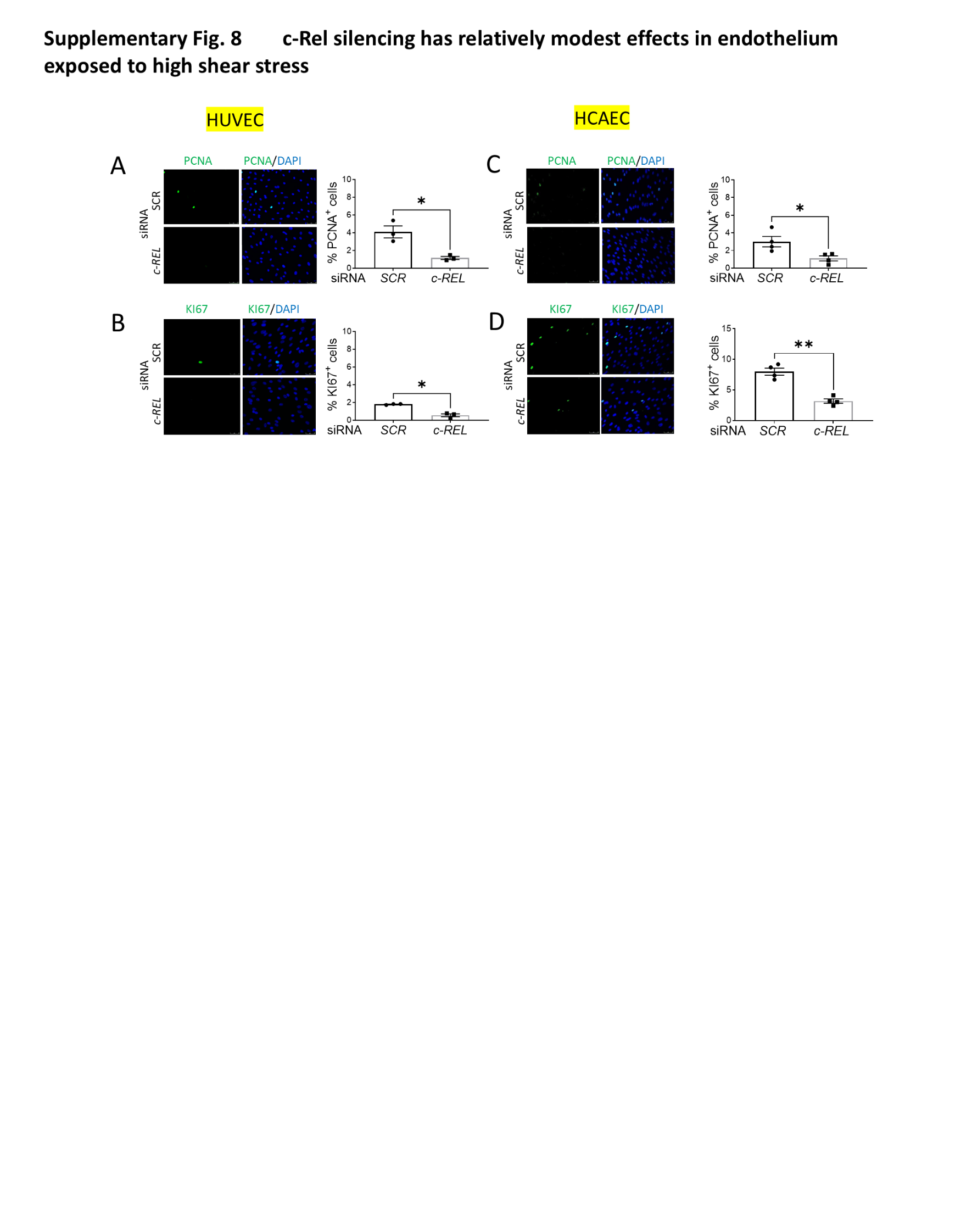
**

**Supplementary Fig. 9: c-Rel silencing has relatively modest effects in endothelium exposed to high shear stress. (A-D)** Human ECs were treated with *c-REL* siRNA or with scrambled non-targeting sequences (SCR) and exposed to high shear stress for 72h using the orbital system. Proliferation was quantified by immunofluorescence staining using antibodies against PCNA (green) in HUVEC **(A)** (n=3 individual donors) or HCAEC (**C**) (n=4 individual donors) and Ki67 (green) in HUVEC **(B)** (n=3 individual donors) or HCAEC (**D**) (n=4 individual donors). Each data point represents average values from 3 fields of view for each donor. Nuclei were co-stained with DAPI (blue) (Scale bar=50 μm). Differences between means were analysed using a paired *t-*test. *P<0.05, **P<0.01.


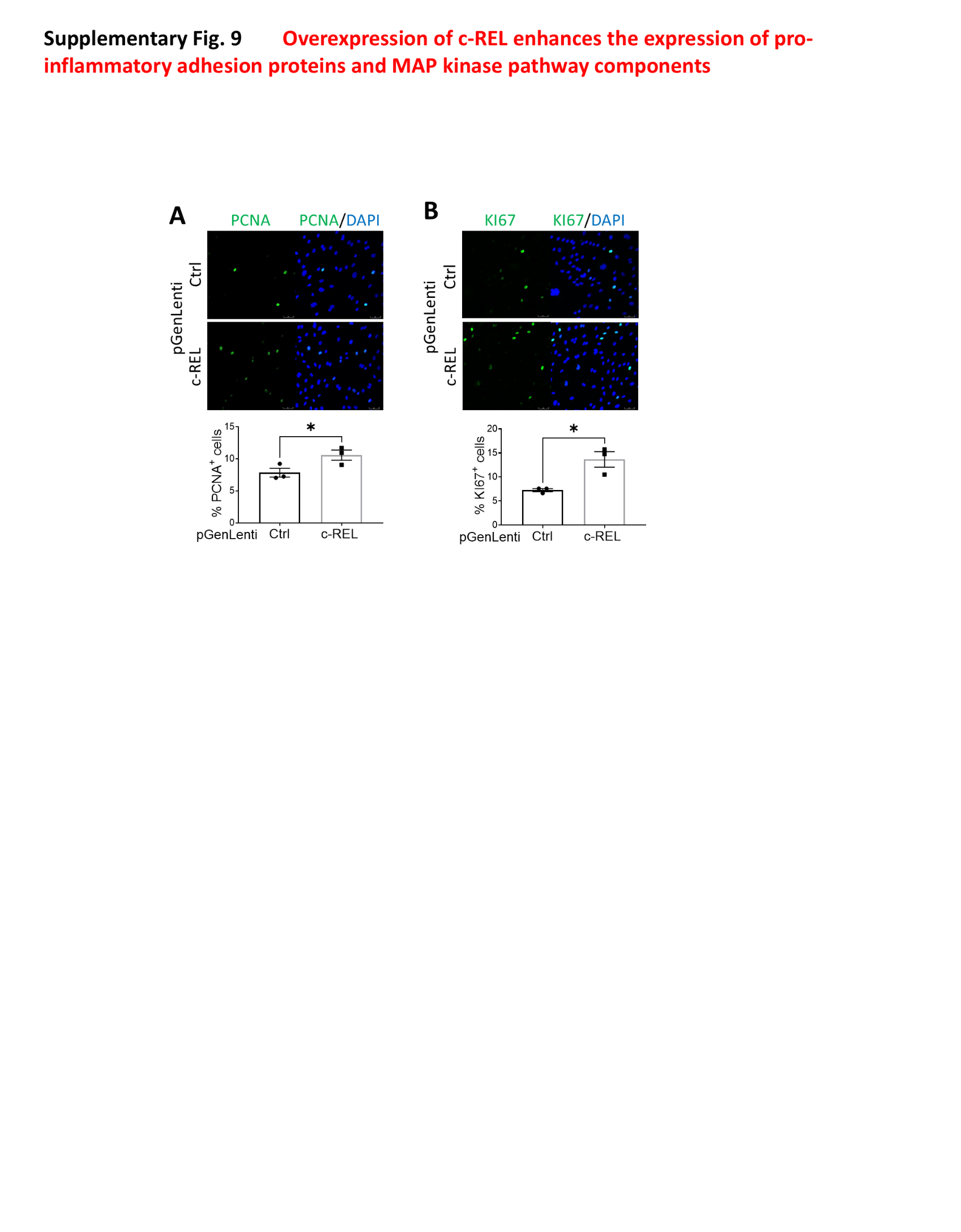


**Supplementary Fig. 10: Overexpression of c-REL enhances the expression of proliferative markers under low shear stress.** HCAECs were transfected with c-REL or control pGenLenti lentiviral vectors and exposed to low shear stress for 72h using the orbital system. Proliferation was quantified by immunofluorescence staining using antibodies against PCNA (**A**) (green) (n=3 individual donors) and Ki67 (**B**) (green) (n=3 individual donors). Each data point represents average values from 3-5 fields of view for each donor. Nuclei were co-stained with DAPI (blue) (Scale bar=50 μm). Differences between means were analysed using a paired *t*-test. *P<0.05.

**
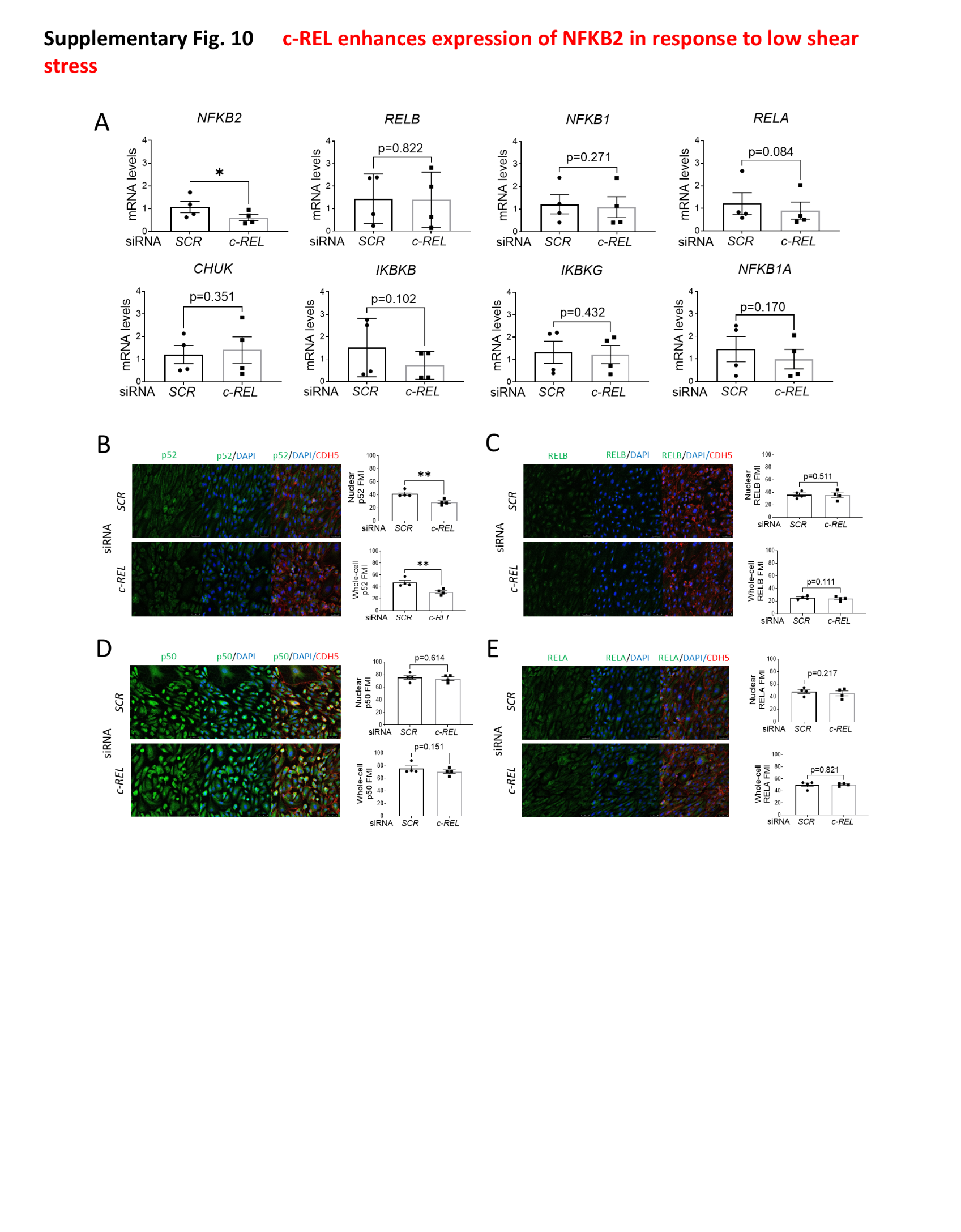
**

**Supplementary Fig. 11: c-REL enhances expression of *NFKB2* in response to low shear stress.** **(A-E)** HCAECs were treated with *c-REL* siRNA or with scrambled non-targeting sequences (SCR) and exposed to low shear stress for 72h using the orbital system. **(A)** Expression levels of different NF-κB subunits and upstream components were quantified by qRT-PCR (n=4 individual donors). **(B-E)** Immunofluorescence staining was performed to quantify levels of p52 (**B**), RELB (**C**), p50 (**D**) and RELA (**E**) (green) (n=4 individual donors). Each data point represents average values from 3-5 fields of view for each donor. Nuclei were co-stained with DAPI (blue) (Scale bar=50 μm). Differences between means were analysed using a paired *t*-test. *P<0.05, **P< 0.01.

**
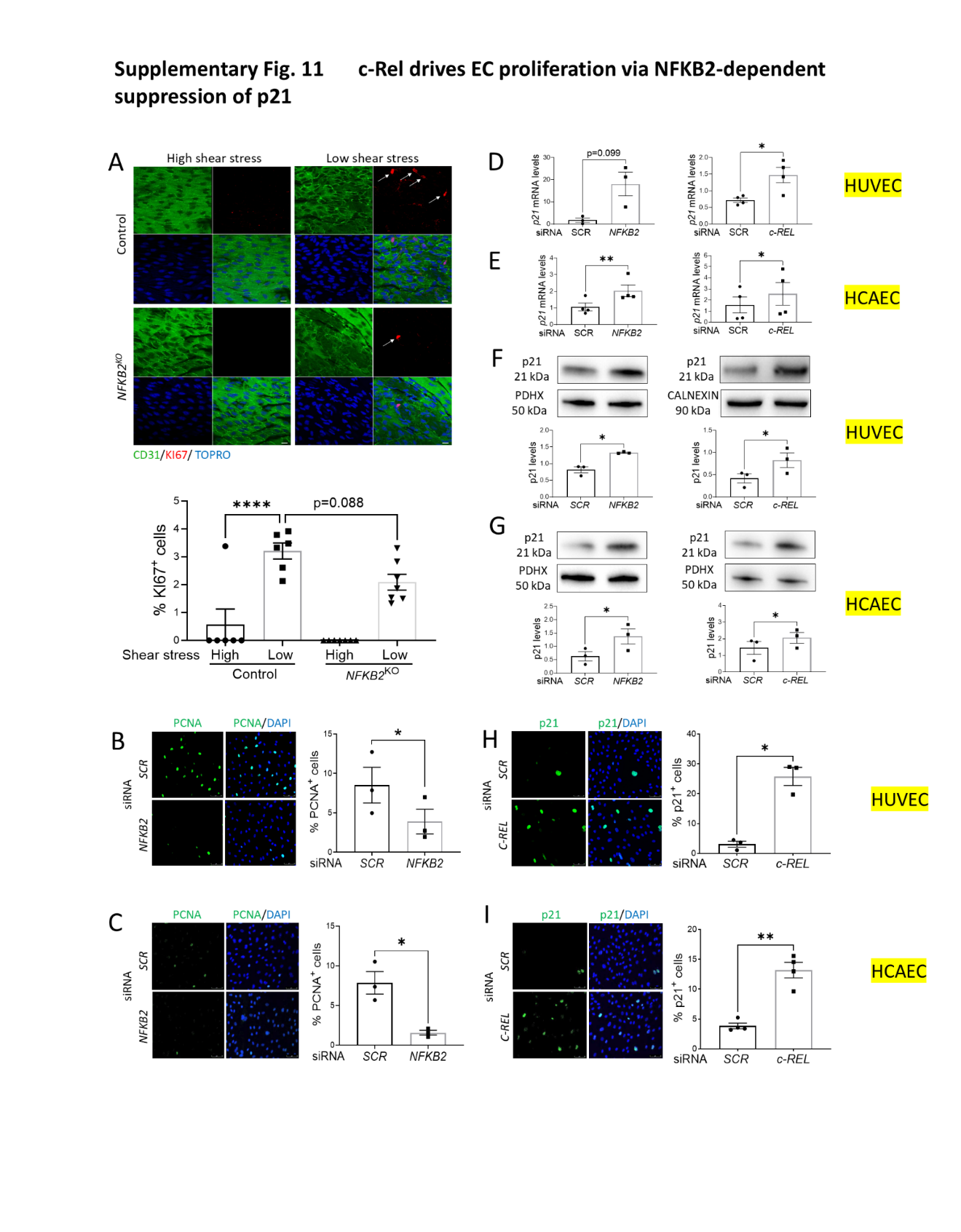
**

**Supplementary Fig. 12: c-Rel drives EC proliferation via *NFKB2*-dependent suppression of p21**. **(A)** Aortic arches from control wild-type mice (n=6) or *NFKB2^KO^* mice (n=7) were analysed by *en face* immunostaining using anti-Ki67 antibodies (red). Endothelium co-stained with anti-CD31 antibodies (green) and TOPRO-3 (DNA; blue). Representative images are shown (Scale bar=10 μm). The proportion of proliferative Ki67-positive cells was calculated by averaging values from 3-5 fields of view and mean values +/- SE are shown. **(B-I)** Human ECs were treated with *NFKB2* siRNA or *c-REL* siRNA or with scrambled non-targeting sequences (SCR) and exposed to low shear stress for 72h. **(B, C)** Proliferation was quantified by immunofluorescence staining using anti-PCNA (green) in HUVEC **(B)** (n=3 individual donors) or HCAEC **(C)** (n=3 individual donors). Nuclei were co-stained with DAPI (blue) (Scale bar=50 μm). The proportion of proliferative PCNA-positive cells was calculated by averaging values from 3-5 fields of view and mean values +/- SE are shown. **(D, E)** *P21* mRNA quantification by qRT-PCR in HUVEC **(D)** (n=3-4 individual donors) or HCAEC **(E)** (n=4 individual donors). **(F, G)** P21 analysis by immunoblotting normalized to PDHX or CALNEXIN in HUVEC **(F)** (n=3 individual donors) or HCAEC **(G)** (n=3 individual donors). **(H, I)** P21 (green) analysis by immunofluorescence staining in HUVEC **(H)** (n=3 individual donors) or HCAEC **(I)** (n=4 individual donors). Nuclei co-stained with DAPI (blue) (Scale bar = 50 μm). The proportion of P21-positive cells was calculated by averaging values from 3-5 fields of view and mean values +/- SE are shown. Differences between means were analysed by 2-way ANOVA **(A)** or using a paired *t-*test **(B-I).** *P<0.05, **P<0.01, ****P<0.0001.


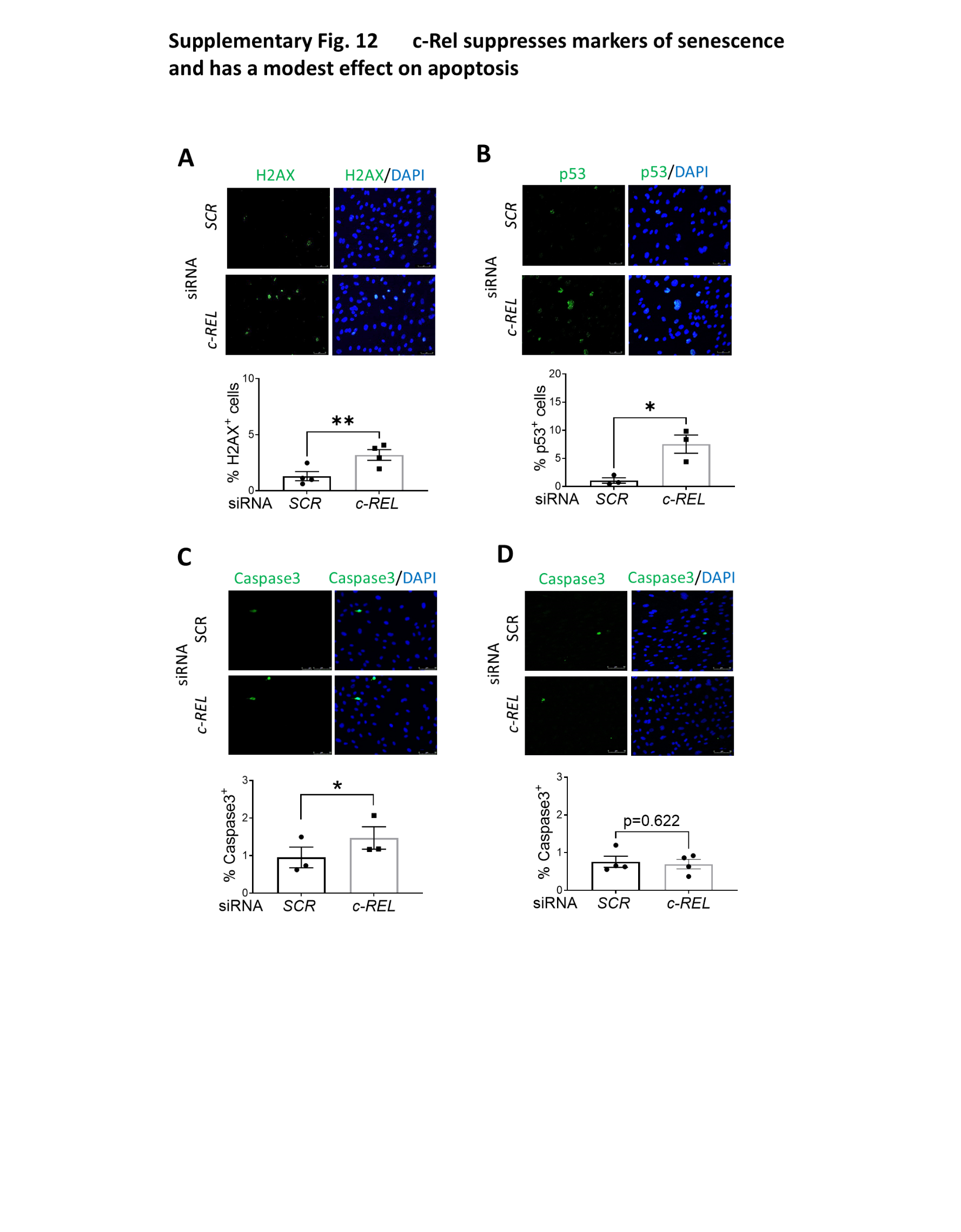


**Supplementary Fig. 13: c-Rel suppresses markers of senescence but has a modest effect on apoptosis.** Human ECs were treated with *c-REL* siRNA or with scrambled non-targeting sequences (SCR) and exposed to low shear stress for 72h. **(A, B)** Senescence was quantified by immunofluorescence staining in HCAEC using antibodies against H2AX **(A)** (n=4 individual donors) and p53 **(B)** (n=3 individual donors) (green). Each data point represents average values from 3-5 fields of view for each donor. Nuclei were co-stained with DAPI (blue) (Scale bar=50 μm). **(C, D)** Apoptosis was quantified by immunofluorescence staining using antibodies against active caspase-3 (green) in HUVEC **(C)** (n=3 individual donors) and HCAEC **(D)** (n=4 individual donors). Each data point represents average values from 3-5 fields of view for each donor. Nuclei were co-stained with DAPI (blue) (Scale bar=50 μm). Differences between means were analysed using a paired *t-*test. *P<0.05, **P<0.01.

**Supplementary Fig. 14: Global *c-Rel* deletion reduces plasma cholesterol and attenuates atherosclerosis. (A, C)** *c-Rel^KO^* mice and controls received one injection of PCSK9-AAV virus and were fed with a Western diet for 6 weeks. **(A)** Representative images of aortas stained with oil Red O and quantification of plaque burden (% aortic surface area with plaque in *c-Rel^KO^* mice (n=10) and control mice (n=10) (Scale bar=2 mm). **(B)** Total plasma cholesterol and triglyceride levels from *c-Rel^KO^* mice (n=9) and controls (n=10) were measured. **(C)** The percentage of microsteatosis, macrosteatosis, and total lipid droplets in formalin-fixed liver tissue stained with hematoxylin-eosin was quantified in *c-Rel^KO^* mice (n=6) and controls (n=4) (Scale bar=100 μm). Blue arrows indicate inflammation and black arrow indicates lipid droplets. (PT: Portal tract, CV: Central vein). **(D)** Expression of hepatic lipid regulators were quantified by qRT-PCR in livers from 16-week-old *c-Rel^KO^* mice (n=4) and control mice (n=4). Mean levels +/- standard errors are shown. Differences between means were analysed using an unpaired *t*-test. *P<0.05, **P<0.01, ***P<0.001.

**
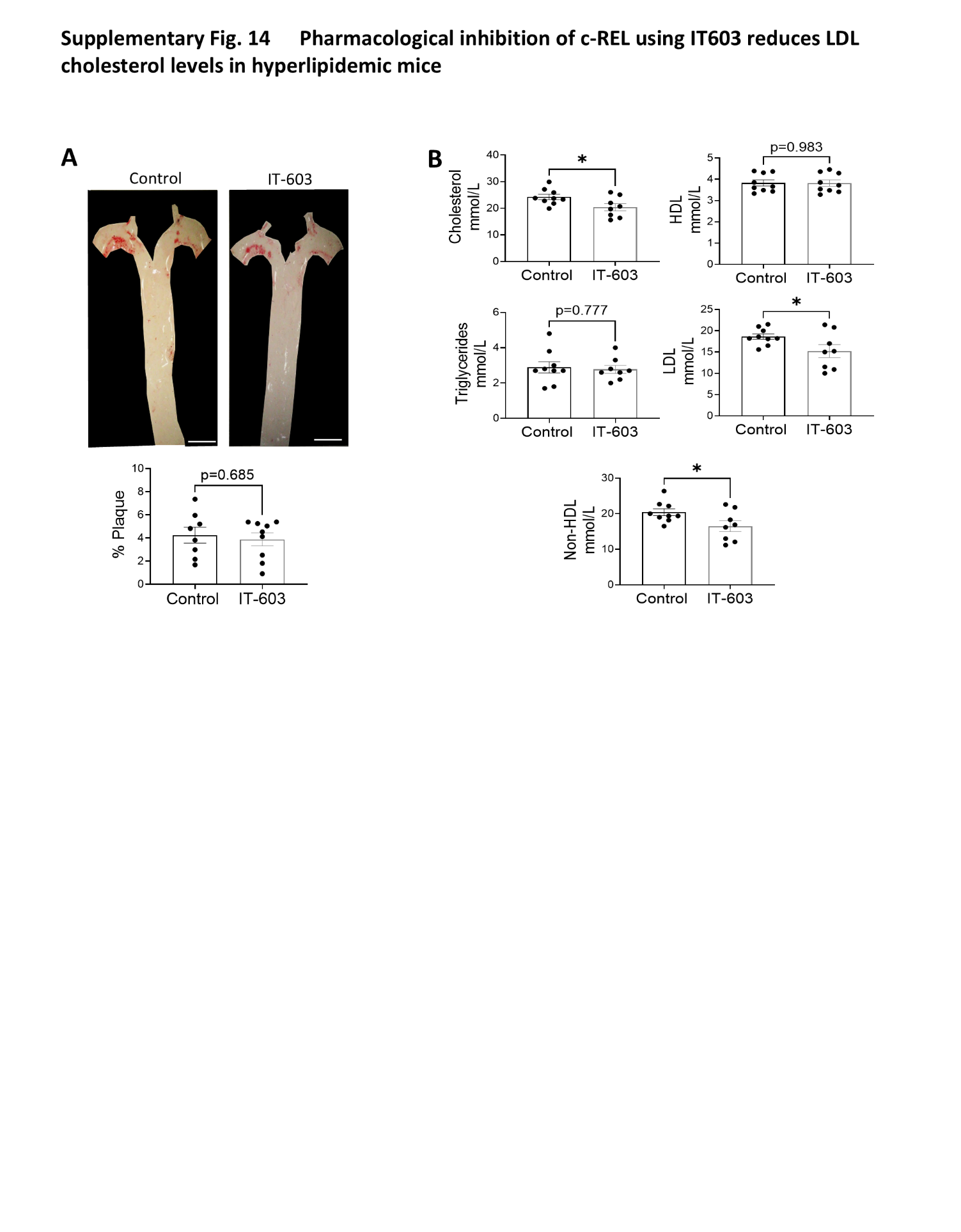
**

**Supplementary Fig. 15: Pharmacological inhibition of c-REL using IT603 reduces LDL cholesterol levels in hyperlipidemic mice. (A, B)** Wildtype C57BL/6J mice received one injection of PCSK9-AAV virus followed by a Western diet for 6 weeks. From weeks 3-6, mice received intraperitoneal injections of IT603 to inhibit c-REL or vehicle control injections (3 injections per week). **(A)** Representative images of aortas stained with oil Red O (upper) and quantification of plaque burden by calculating the percentage of aortic surface area covered by plaque (lower) in mice treated with IT-603 (n=9 mice) or control vehicle (n=8 mice) (Scale bar=2 mm). **(B)** Total plasma cholesterol and triglyceride levels in plasma from mice treated with IT-603 (n=8-9 mice) or control vehicle (n=9 mice) were measured. Mean levels +/- standard errors are shown. Differences between means were analysed using an unpaired *t-*test. *P<0.05.

**
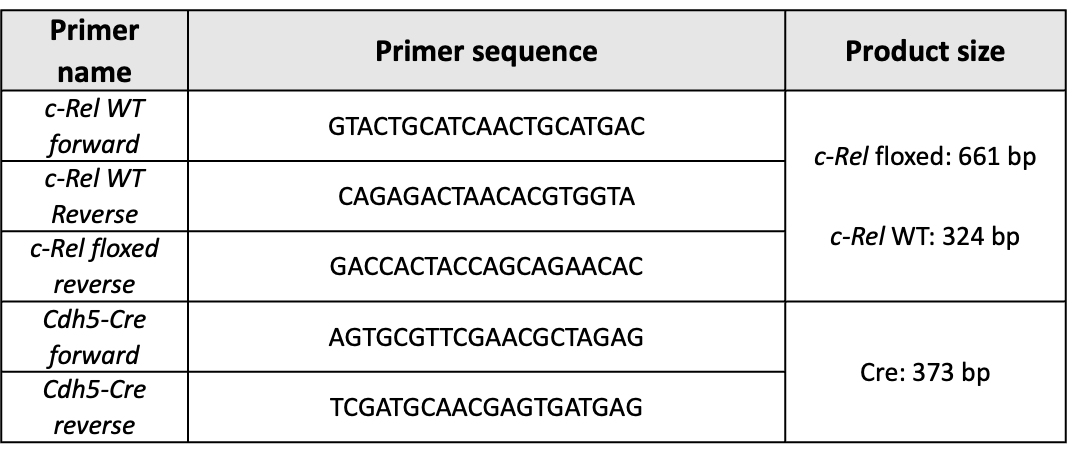
**

**Supplementary Table 1**

**
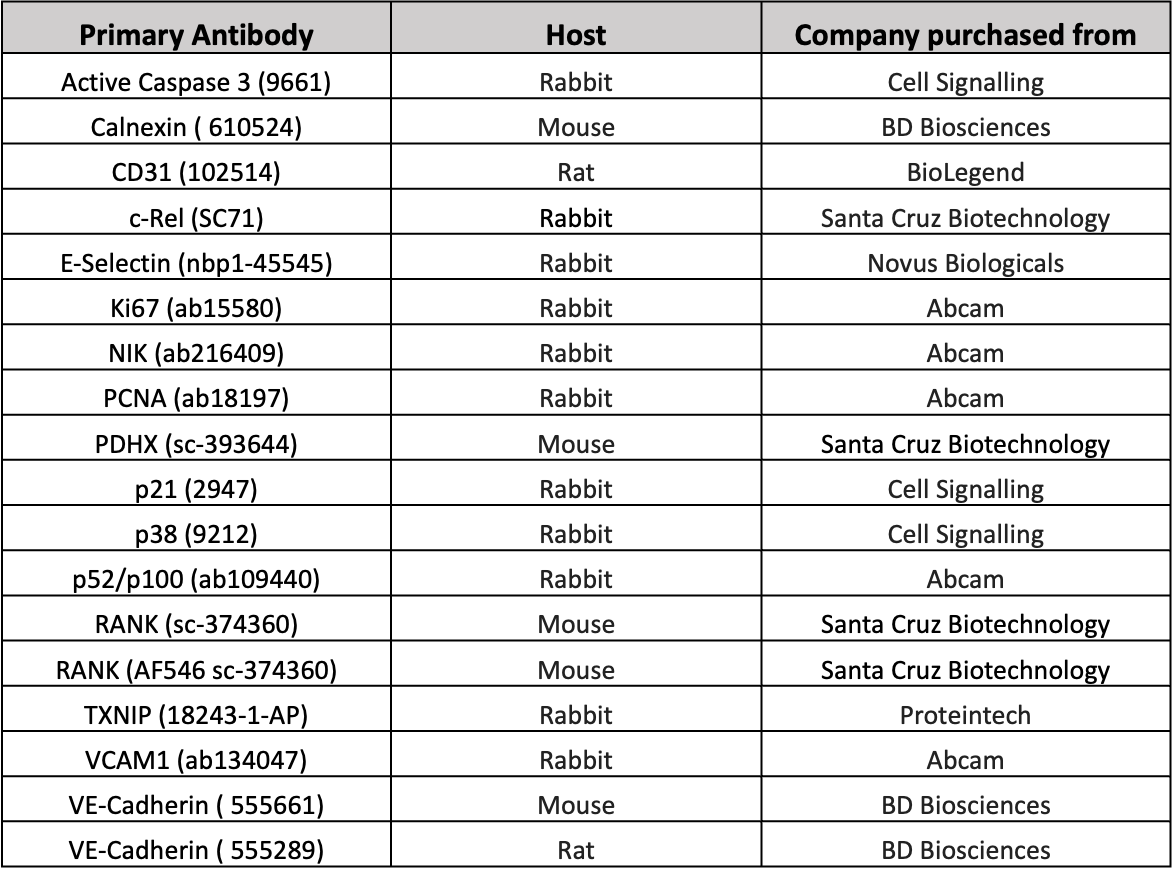

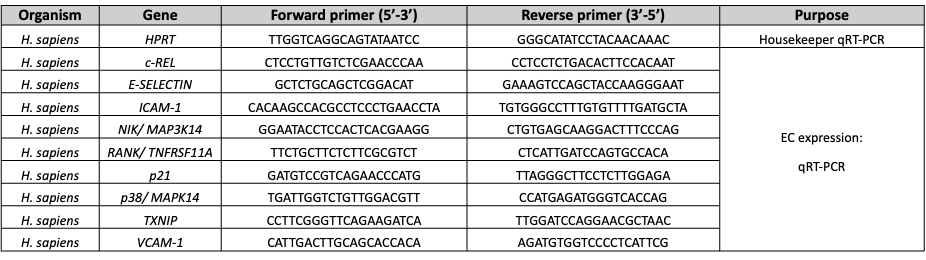
**

**Supplementary Table 2**

**Supplementary Table 3**

**REFERENCES FOR SUPPLEMENTAL MATERIAL**

1. Kan H, Zhang K, Mao A, Geng L, Gao M, Feng L, You Q, Ma X. Single-cell transcriptome analysis reveals cellular heterogeneity in the ascending aortas of normal and high-fat diet-fed mice. *Experimental & Molecular Medicine*. 2021;53:1379-1389. doi: 10.1038/s12276-021-00671-2

2. Kalluri AS, Vellarikkal SK, Edelman ER, Nguyen L, Subramanian A, Ellinor PT, Regev A, Kathiresan S, Gupta RM. Single-Cell Analysis of the Normal Mouse Aorta Reveals Functionally Distinct Endothelial Cell Populations. *Circulation*. 2019;140:147-163. doi: 10.1161/circulationaha.118.038362
